# *REEP1* Accumulation Disrupts ER Integrity and Drives Spinal Motoneuron Degeneration in Distal Hereditary Motor Neuropathy

**DOI:** 10.1002/advs.202511483

**Published:** 2025-11-21

**Authors:** Andrea Bock, Mona Schurig, Miles Willoughby, Andrea Mirecki, Eric Seemann, Kateryna Lohachova, Istvan Katona, Sonnhild Mittag, Lutz Liebmann, Patricia Franzka, Mehdi Heidari Horestani, Mukhran Khundadze, Thorsten Mosler, Timothy Louie, Marianne de Visser, Marian A. J. Weterman, Michael Kiehntopf, Christian Beetz, Sandor Nietzsche, Otmar Huber, Joachim Weis, Michael M. Kessels, Ramachandra M. Bhaskara, Britta Qualmann, Ivan Đikić, Christian A. Hübner

**Affiliations:** ^1^ Institute of Human Genetics Jena University Hospital Friedrich Schiller University Am Klinikum 1 07747 Jena Germany; ^2^ Institute of Biochemistry II Goethe University School of Medicine Theodor‐Stern‐Kai 7 60590 Frankfurt am Main Germany; ^3^ Institute for Biochemistry I Jena University Hospital Friedrich‐Schiller‐University Nonnenplan 2‐4 07743 Jena Germany; ^4^ Buchmann Institute for Molecular Life Sciences Goethe University Frankfurt Max‐von‐Laue Straße 15 60438 Frankfurt am Main Germany; ^5^ Institute of Neuropathology RWTH Aachen University Hospital Pauwelsstr. 30 52074 Aachen Germany; ^6^ Department of Neurology Houston Methodist Research Institute Houston Texas USA; ^7^ Institute for Biochemistry II Jena University Hospital Friedrich‐Schiller‐University Nonnenplan 2‐4 07743 Jena Germany; ^8^ Swift Institute – Vein and Neurology 10381 Double R. Blvd. Reno NV 89521 USA; ^9^ Department of Neurology Academic Medical Center Amsterdam The Netherlands; ^10^ Department of Genome Analysis/Clinical Genetics Amsterdam University Medical Center Location Academic Medical Center Amsterdam 1081 BT The Netherlands; ^11^ Dept Clinical Genetics LUMC Leiden 2300RC The Netherlands; ^12^ Institute for Clinical and Laboratory Medicine Jena University Hospital Friedrich Schiller University Am Klinikum 1 07747 Jena Germany; ^13^ Center for Electron Microscopy Jena University Hospital Ziegelmühlenweg 1 07743 Jena Germany; ^14^ Center for Rare Diseases Friedrich Schiller University Am Klinikum1 07747 Jena Germany; ^15^ Present address: Institute of Diagnostic Laboratory Medicine Clinical Chemistry and Pathobiochemistry Charité – Universitätsmedizin Berlin 13353 Berlin Germany

**Keywords:** dHMN, endoplasmic reticulum, HSP, membrane shaping, *REEP1*, ubiquitination

## Abstract

*REEP1* contributes to the shaping of the endoplasmic reticulum (ER) through conserved transmembrane hairpins and a long C‐terminal amphipathic helix. *REEP1* loss‐of‐function causes hereditary spastic paraplegia due to degeneration of cortical motoneuron axons. Patients with deletion of *REEP1* exon5 (Δexon5), which deletes part of its amphipathic helix, however, develop muscle atrophy due to degeneration of spinal motoneuron axons (distal hereditary motor neuropathy/dHMN). It is known that *REEP1* knockout mice exhibit simplified ER structures in cortical motoneurons. Here, we show that these neurons are progressively lost while spinal motoneurons remain intact. Conversely, Δexon5 knockin (KI) mice lose spinal motoneurons preceded by ER fragmentation, whereas cortical motoneurons remain intact. Mechanistically, *REEP1* undergoes ubiquitination and proteasomal degradation, a process compromised in the Δexon5 variant due to impaired ubiquitination, which thus accumulates in peripheral nerves. Proteomic analysis identifies *HUWE1* as the E3 ligase responsible for *REEP1* turnover. Modeling and liposome shaping assays reveal that the Δexon5 variant retains its capacity to induce membrane curvature. Consistently, other *REEP1* variants associated with dHMN also show compromised ubiquitination and preserved transmembrane hairpins. Therefore, it is proposed that accumulation of shaping‐competent *REEP1* variants in the ER drives ER fragmentation and spinal motoneuron degeneration in dHMN.

## Graphical abstract

*REEP1* contributes to endoplasmic reticulum (ER) shaping. Variants either cause cortical motoneuron degeneration and hereditary spastic paraplegia (HSP) or spinal motoneuron degeneration and distal hereditary motor neuropathy (dHMN). Knockout  causes less complex ER structures and cortical motoneuron loss. dHMN knockin causes accumulation of the variant protein due to impaired ubiquitination and proteasomal degradation, thus causing ER‐fragmentation and spinal motoneuron loss.

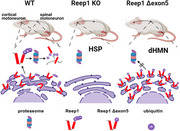

## 1 Introduction

The endoplasmic reticulum (ER) is the largest organelle of the cell. Its functions include cotranslational synthesis of membrane and secretory proteins, lipid and lipid droplet synthesis, Ca^2+^ storage, membrane supply for autophagosomes, and more.^[^ ^1^, ^2^ ^]^ Neurons require high rates of membrane protein and lipid synthesis processes because of their extensive surface area and large cytoplasmic volume. Therefore, the ER is a central hub for neuronal function, which extends into distal compartments including dendrites and axons.^[^ ^3^ ^]^ In axons, it consists of mostly elongated tubules with few branch points often associated with sheets.^[^ ^4^, ^5^ ^]^

The importance of the ER for neuronal maintenance is highlighted by numerous neurodegenerative disorders, which are caused by variants in genes encoding ER proteins. Several encode membrane‐shaping proteins, and their loss‐of‐function primarily results in the degeneration of particularly long axons, also known as axonopathies.^[^ ^6^ ^]^ The degeneration can primarily affect afferent or efferent axons and thus predominantly result in sensory or motor deficits. If mainly corticospinal tract fibers, i.e., the axons projecting from cortical motoneurons to spinal cord motoneurons, degenerate, this causes spasticity and muscle weakness of the legs as observed in hereditary spastic paraplegia (HSP). While pure HSP does not show additional symptoms, symptoms in complicated HSP can also include e.g. mental retardation, epilepsy, cerebellar ataxia, or optic atrophy.^[^ ^7^ ^]^ SPG31 is one of the most common forms of dominant pure HSP without muscular atrophy or sensory symptoms and is caused by loss‐of‐function variants in *REEP1*.^[^ ^8^ ^]^ Surprisingly, however, the *REEP1* splice site variant c.304‐2A>C, which leads to the deletion of *REEP1* exon 5 (Δexon5), is associated with distal hereditary motor neuropathy (dHMN), which is characterized by slowly progressive distal‐limb‐muscle weakness and wasting without spasticity.^[^ ^9^ ^]^

The REEP family of proteins comprises two branches in vertebrates (*REEP1*–*4* and *REEP5*, *6*) with a single ancestral protein in yeast (*Yop1*).^[^ ^10^, ^11^ ^]^ *REEP5* and *REEP6* are characterized by four transmembrane (TM) segments followed by a conserved C‐terminal amphipathic helix (AH). Each pair of adjacent TM helices fold into a transmembrane hairpin‐like structure, forming a wedge‐like structure that induces high local membrane curvature upon membrane insertion, a feature shared by REEPs and other evolutionarily related Reticulon proteins (RTNs).^[^ ^12^, ^13^ ^]^ Therefore, these membrane domains are also known as Reticulon homology domains (RHDs). In humans, *REEP1*–*4* lack the first transmembrane segment, while the remaining structural elements (TM2–4 and AH) are still preserved. *REEP1* and homologs have been linked to different functions such as ER morphogenesis,^[^ ^10^ ^]^ lipid droplet formation,^[^ ^14^ ^]^ mitotic ER dynamics,^[^ ^15^ ^]^ nuclear pore formation,^[^ ^16^ ^]^ and autophagy in fission yeast.^[^ ^17^, ^18^, ^19^ ^]^ More recently, *REEP1* was identified as being enriched in a unique vesicular ER‐derived compartment.^[^ ^20^ ^]^ Although these vesicles originate from the bulk ER, their precise functions remain unknown. They are recycled by fusing with ER subdomains. This process requires *REEP1* to form a physical complex with Atlastins, membrane‐bound GTPases,^[^ ^20^ ^]^ which mediate the tethering and fusion of ER tubules to form the three‐way junctions of the polygonal ER network.^[^ ^21^, ^22^ ^]^

Despite extensive cellular and biochemical characterization of *REEP1*, the molecular mechanisms leading to the pathogenesis of HSP and dHMN remain largely unclear. We have previously reported that homozygous *REEP1* knockout (KO) mice reproduce HSP with a length‐dependent degeneration of corticospinal tract fibers, thus establishing *REEP1* loss‐of‐function and haploinsufficiency as causative for SPG31.^[^ ^23^ ^]^ We have now modeled the dHMN‐associated *REEP1* splice‐site variant c.304‐2A>C, which causes the in‐frame deletion of exon 5 of *REEP1* in a knockin (KI) mouse model. By comparing both mouse models, we show a pathology of cortical motoneurons in *REEP1* homozygous KO (KO/KO) mice, which is absent in homozygous KI mice. By contrast, *REEP1* homozygous KI (KI/KI) mice display a degeneration of spinal motoneurons, which is not observed in KO/KO mice. Individual ER structures were longer but fewer in number in cortical motoneurons of KO/KO mice, while the ER structure appeared intact in cortical motoneurons of KI/KI mice. In KI/KI mice, however, the ER structures were shorter and more abundant in spinal motoneurons, whereas the ER structure appeared intact in KO/KO spinal motoneurons. Notably, membrane shaping properties of the Δexon5 variant were preserved in vitro, consistent with our modeling and molecular dynamics simulations in model bilayers. While the *REEP1* wild‐type (WT) protein was strongly ubiquitinated and degraded via the proteasome, decreased ubiquitination was a consistent feature of the Δexon5 variant due to the loss of critical ubiquitination sites. As the abundance of the Δexon5 variant was strongly increased in spinal cord and sciatic nerve lysates of KI mice, we propose that a reduced turnover leads to an overload of shaping‐competent aberrant *REEP1* in the ER of spinal motoneurons. The preserved membrane remodeling capacity consequently leads to ER fragmentation and neuron loss. In agreement, we found that other variants associated with dHMN also have intact RHDs and exhibit compromised ubiquitination in vitro.

## 2 Experimental Section

### 2.1 Mice

All animal studies were approved by the “Thüringer Landesamt für Verbraucherschutz” (TLV) and performed in accordance with the regulation of animal welfare (approval numbers: 02‐017/15 and UKJ‐17‐006). Mice were bred and housed in single‐ventilated cages with a 12 h light/dark cycle under specific pathogen‐free conditions and fed on a regular diet ad libitum at the animal facility of the Jena University Hospital. Mice were genotyped from DNA isolated from tail biopsies or ear punches by polymerase chain reaction (PCR) (for primers see table) and maintained on a C57BL/6J background. The KO line was genotyped by two separate PCRs, either amplifying the WT or the KO allele (*REEP1* WT F/R and *REEP1* KO F/R). The KI line was genotyped with a 3‐primer PCR (*REEP1* dHMN F with *REEP1* dHMN KI R and *REEP1* dHMN WT R).

The dHMN‐associated variant c.3042A>G altered the splice acceptor of exon 5, thus leading to the skipping of exon5.^[^ ^9^ ^]^ To model this variant in mice, two large PCR fragments were cloned using genomic C57BL/6J DNA as a template in the V901 plasmid. A loxP site was cloned into intron 4 and a Neomycin selection cassette flanked by loxP sites at the 3′ end was cloned into intron 5. After electroporation of the linearized construct into R1 embryonic stem cells and positive selection, several clones were derived, which were screened for homologous recombination by PCR. After transient expression of Cre‐recombinase, correctly targeted clones were identified by PCR and subsequently injected into donor blastocysts, which were then transferred into foster mice. Chimeric mice were used to generate heterozygous offsprings, which were subsequently mated to obtain mice with a homozygous deletion of exon5,. Genotyping was performed on DNA isolated from ear punches by PCRs either amplifying the WT or the recombinant allele using the primers in **Table** **1**. Mice were back‐crossed for at least five generations. The KO line was already previously reported.^[^ ^23^ ^]^ Experimenters were blinded for genotypes wherever possible. Animal cohorts were age and sex matched and preferentially selected from littermates. Mice were either sacrificed by decapitation or by perfusion fixation after deep anesthesia with an intraperitoneal injection of a mixture of Xylazine and Ketamine.

**Table 1:** Genotyping primers.

Primer name	Sequence 5′–3′
*REEP1* dHMN F	TGA GGG AGG ACG ATG GTG AC
*REEP1* dHMN WT R	GAT GGG TTA GCT ATT GCC AAG
*REEP1* dHMN KI R	CTT TAT TTT CAT GAT CTG TGT GTT GG
*REEP1* WT F	CTG CAG GCT TAT ATT TGG CAC CCT TTA TCC TGA ATA TTA TTC ATA CAA GG
*REEP1* WT R	CCC GGG GAT ATC GGC GCG CCT GAG GGA ACT GGC CAG AGA G
*REEP1* KO F	TTA AAA ATA CCT ATT AGG CTG TG
*REEP1* KO R	GGA AGA AGG TGG TCT GTG

### 2.2 Mouse Phenotyping

#### 2.2.1 Beam Walk Test

Mice were placed on an elevated beam of 1 m length and 4 cm width with the home cage at the end. After habituation on three consecutive days, the mouse was videotaped from behind while walking on the beam. The foot base angle of the hind limb was measured at the moment when the toe was lifted.

#### 2.2.2 RotaRod Analysis

Mice were placed on a rotating rod (4 rpm) for 2 min, which was then continuously accelerated (up to 40 rpm within 5 min). The latency was determined until the mouse fell off the rod.

#### 2.2.3 Kondziella's Inverted Screen Test

Mice were placed in the center of a wire mesh (1 mm wire with 8 mm squares) that was inverted. The latency until the animals fell off the grid was measured.

#### 2.2.4 Electrophysiological Analysis of Peripheral Nerves

Anesthetized mice (100 mg kg^−1^ ketamine, 16 mg kg^−1^ xylazine) were placed on a heating pad. One pair of needle electrodes with a tip distance of 5 mm (WE30030.1H10, Science Products) was inserted near the base of the tail and a second pair was 30 mm distal to the stimulation site close to the tip of the tail. For the analysis of motor fibers, the stimulus was applied via the proximal electrodes and the response was recorded with the distal electrodes. Compound muscle action potentials (CMAPs) and sensory nerve action potentials were evoked with increasing intensity (0–15 V, increment 1 V, 50 µs duration, interstimulus interval 20 s). Sum action potentials were filtered (high‐pass filter 3 Hz, low‐pass filter 1.3 kHz) and digitized with a sampling frequency of 10 kHz. Amplitudes were determined from peak to peak.

### 2.3 Cloning

Cloning and amplification of plasmids were performed in *Escherichia coli* XL1 blue cells (200249, Agilent). The human *REEP1* cDNA sequence was cloned into the pCS2+ expression plasmid. Mutations were introduced by site‐directed mutagenesis by a two‐step PCR and subsequently verified by Sanger sequencing.

### 2.4 Cell Culture

HEK‐293T and HeLa cells were provided by the American Type Culture Collection (Manassas, VA). Their identities were authenticated by STR analysis. HeLa‐TRex cells were provided by Prof. S. Taylor (Manchester University). All cell lines were regularly tested for mycoplasma contamination using the LookOut Mycoplasma PCR Detection Kit (Sigma‐Aldrich). Cells were maintained at 37 °C with 5% CO_2_ in Dulbecco's modified Eagle medium (Gibco) supplemented with 10% v/v fetal bovine serum (Gibco) and 100 U mL^−1^ penicillin and streptomycin (Gibco). Inducible cell lines were induced with 1 µg mL^−1^ doxycycline (Sigma‐Aldrich). Bafilomycin A1 (LC‐Laboratories) was used at a concentration of 200 ng mL^−1^ and MG‐132 (Sigma‐Aldrich) at 15 µm. DNA plasmids were transfected with Lipofectamine 2000 (Invitrogen) for transient expression.

For the culture of primary neurons, P1 pups were decapitated and brains dissected in ice‐cold HBSS (Thermo Fisher) with 1% pen/strep and 7 mm *N*‐(2‐hydroxyethyl)piperazine‐29‐(2‐ethane‐sulfonic acid) (HEPES). Hippocampi were rinsed thrice with HBSS and trypsinized with 0.05% trypsin/ethylenediaminetetraacetic Acid (EDTA) (Invitrogen) for 25 min at 37 °C. The supernatant was removed and rinsed with HBSS. The tissue was placed in 2 mL of HBSS containing 20 µL of DNase (1 µg µL in HBSS) and dissociated into single cells by trituration with folded Pasteur pipettes of decreasing diameter. Approximately 50 000 cells were plated onto poly‐l‐lysine (Sigma‐Aldrich)‐coated 18 mm coverslips in plating medium (minimum essential medium, 10% v/v horse serum, 0.6% w/v glucose). Cells were allowed to settle for 10–20 min at 37 °C and 5% CO_2_. Finally, coverslips were transferred to 6 cm culture dishes containing Neurobasal medium (Invitrogen) supplemented with 1 mm l‐glutamine, 0.2% v/v horse serum, and B27 supplement (Gibco). Neurobasal medium supplemented with B27 was added weekly to maintain the initial volume.

### 2.5 Quantitative Real‐Time PCR

RNA was isolated from dissected brain or spinal cord tissues using TRIZOL. cDNA synthesis was performed using 2 µg of total RNA with the GoScript reverse transcription kit. qPCR was performed in triplicate on a 9‐well plate in a CFX96 real‐time cycler (Bio‐Rad). The primer sequences are given in **Table** **2**. Relative *REEP1* transcript abundance was calculated between *REEP1* ^WT^ and *REEP1* ^KI/KI^ brains and spinal cords using CFX Maestro software (Bio‐Rad). Actin and GAPDH levels served as housekeeping controls.

**Table 2:** Primers for real‐time PCR.

Primer name	Sequence 5′–3′
Rp1 F	CAT ACA AGG CTG TGA AGT CCA AG
Rp1 R	GGA AAC CAG CAA AGG AAG ATG TC
Actin F	AGA GGG AAA TCG TGC GTG AC
Actin R	CAA TAG TGA TGA CCT GGC CGT
GAPDH F	AAC TTT GGC ATT GTG GAA GG
GAPDH R	ACA CAT TGG GGG TAG GAA CA

### 2.6 Western Blot Analysis

To determine the abundance of specific proteins, tissue and cell lysates were separated by sodium dodecyl sulfate‐polyacrylamide gel electrophoresis (SDS‐PAGE) and transferred to polyvinylidene difluoride membranes. Membranes were blocked for 1 h with 2% w/v bovine serum albumin (BSA) in Tris‐buffered saline‐Tween 20 (TBS‐T) followed by primary antibodies diluted in 1% w/v BSA in TBS‐T overnight at 4 °C. The following day, the membranes were washed 3 times for 10 min with TBS‐T and incubated with the respective secondary antibody diluted in TBS‐T for 2 h at room temperature (RT). Enhanced chemiluminescence detection solutions (Bio‐Rad) were mixed in a 1:1 ratio. Membranes were incubated with the mixture for 2 min and signals detected with the LAS 4000 system (GE Healthcare). Antibodies are provided in Table 3 and 4.

### 2.7 Ubiquitination Assays

HEK‐293T cells were cotransfected with FLAG‐tagged *REEP1* constructs in pCS2+ and His_6_–ubiquitin in pCI. After 24 h, cells were washed with ice‐cold phosphate‐buffered saline (PBS). Subsequently, 200 µL imidazole lysis buffer (20 mm imidazole pH 8.0, 150 mm NaCl, 2 mm MgCl_2_, 300 mm sucrose, and 0.25% v/v Triton X‐100) was added and cells were collected. For cell lysis, tubes were incubated on ice for 10 min followed by centrifugation for 15 min at 14 000 *g* at 4 °C. 10% of the supernatant was mixed with 6× SDS sample buffer (30% v/v 2‐mercaptoethanol, 40% v/v glycerol, 6% w/v SDS, Tris/HCl, pH 6.8) and stored at −20 °C. The remaining supernatant was diluted with 200 µL of lysis buffer. Ni‐NTA beads (Qiagen) were washed with lysis buffer and added to each lysate. The tubes were shaken for 1 h at 4 °C. Then, the beads were collected by centrifugation for 3 min at 2700 *g* and the supernatant removed. The beads were washed 6 times with 300 µL of lysis buffer each. Finally, 45 µL 2× SDS sample buffer was added, and the beads were boiled for 10 min at 95 °C. Tubes were shortly vortexed and stored at −20 °C. The next day, samples were analyzed by SDS‐PAGE (**Table** 3, 4, 5). Antibodies are provided in Table 3 and 4.

**Table 3:** Primary antibodies.

Antibody	Host species	Dilution IF	Dilution WB	Product number, company
α‐Bungarotoxin Alexa Fluor 555 conjugate	–	1:500	–	B35451, Invitrogen
*ATL1*	Rabbit	–	1:1000	12728, Cell signaling
CD68	Rabbit	1:500	–	MAB10114‐SP, Biotechne
Beta actin	Mouse	–	1:10 000	ab6276, Abcam
Beta actin	Rabbit	–	1:1000	20536‐1‐AP, Proteintech
Beta III tubulin	Rabbit	–	1:1000	T2200, Sigma‐Aldrich
FLAG	Rabbit	–	1:5000	F7425, Sigma
GAPDH	Rabbit	–	1:1000	sc‐25778, Santa Cruz
GFP	Rabbit	–	1:5000	Ab6556, Abcam
Hoechst 33258	–	1:1000	–	H3569, Invitrogen
*HUWE1*	Rabbit	–	1:1000	19430‐1‐AP, Proteintech
Laminin	Rabbit	1:1000	–	ab11575, Abcam
Lamp1 (CD107a)	Rat	1:250	–	553792, BD Biosciences
Lamp1	Mouse	–	1:1000	sc‐20011, SantaCruz
LC3B	Rabbit	1:1000	1:1000	2775S, Cell Signaling
NeuN	Mouse	1:300	–	MAB377, Merck Millipore
Neurofilament 200	Mouse	1:250	–	N0142, Sigma
p62/SQSTM1	Mouse	1:1000	1:1000	ab56416, Abcam
*REEP1*	Rabbit	–	1:500	17988‐1‐AP, Proteintech
*REEP2*	Rabbit	–	1:1000	15684‐1‐AP, Proteintech
*REEP3*	Rabbit	–	1:1000	Ab106463, Abcam
*REEP4*	Rabbit	–	1:1000	26650‐1‐AP, Proteintech
SMI312	Mouse	–	1:500	837904, Biolegend
Ubiquitin	Mouse	–	1:500	13‐1600, Thermo Fisher Scientific
Ubiquitin	Rabbit	–	1:500	10201‐2‐AP, Proteintech
Vinculin	Rabbit	–	1:1000	14‐9777‐82, Invitrogen

**Table 4:** Secondary antibodies.

Antibody	Dilution IF	Dilution WB	Product number, company
Goat anti‐Mouse IgG, Alexa Fluor 488	1:500, 1:1000	–	A11029, Thermo Fisher
Goat anti‐Rabbit IgG, Alexa Fluor 488	1:1000	–	A11008, Thermo Fisher
Goat anti‐Mouse IgG, Alexa Fluor 546	1:1000	–	A11030, Thermo Fisher
Mouse IgG HRP Linked F(ab′)2	–	1:10 000	GENA9310, Merck
Rabbit IgG HRP Linked F(ab′)2	–	1:10 000	GENA9340, Merck

**Table 5:** Summary of systems used in molecular dynamics simulations. Details of the protein, dimensions of cubic simulation boxes, number of POPC lipids in the upper (*N*_U_) and lower leaflets (*N*_L_), number of CG water beads and ions, and total simulation time are provided for each system.

Protein *REEP1*	Simulation box [*a* × *b* × *c* nm^3^]	#. *N*_U_	#. *N*_L_	#. CG water	#. Na^+^	#. Cl^−^	Time [µs]
WT (x1)	40 × 40 × 20	2675	2698	197 360	2891	2900	10
Δexon5 (x1)	40 × 40 × 20	2681	2696	197 385	2891	2900	10
WT (x2)	40 × 40 × 20	2647	2687	197 129	2891	2909	10
Δexon5 (x2)	40 × 40 × 20	2666	2690	197 199	2891	2909	10

For the siRNA‐mediated knockdown of *HUWE1*, HEK‐293T cells were transfected with the siRNA (Horizon Dharmacon, #J‐007185‐09‐0002) at a final concentration of 10 nm using Lipofectamine 2000. The following day, cells were transfected with *REEP1*‐WT, His_6_–ubiquitin, or His_6_‐expression vectors. After 24 h, the cells were lysed and analyzed by Western blot.

### 2.8 Cross‐Linking

HEK‐293T cells were transfected with various *REEP1* constructs in the pCS2+ vector. 24 h later, plates were washed with ice‐cold PBS. Then, 200 µL cross‐linking lysis buffer was added, and the cells were collected. For cell lysis, tubes were incubated on ice for 30 min, followed by centrifugation at 14 000 *g* and 4 °C for 30 min. For the cross‐linking, 100 µL of 0.025% glutaraldehyde was added and incubated for 30 min at 4 °C. The reaction was stopped with 100 mm glycine. Cross‐linked and untreated samples were mixed with 2× SDS sample buffer and analyzed by SDS‐PAGE.

### 2.9 Counting of Cortical Motoneurons

Mice were deeply anesthetized and perfused transcardially with PBS (pH 7.4) followed by 4% w/v paraformaldehyde (PFA) in PBS for 10 min. After dissection, tissues were postfixed in 4% w/v PFA in PBS for at least 1 h. Then, tissues were incubated in sucrose (10% w/v sucrose for 4 h and in 30% w/v sucrose overnight at 4 °C) and cut with a cryotome. Sections of 10 µm were mounted on glass slides. After washing with PBS, slides were transferred to 90 °C hot citric acid buffer (1.8 mm citric acid, 8.2 mm trisodium citrate dihydrate) for 30 min, and subsequently incubated on ice for 15 min. Sections were washed with PBS and permeabilized with PBS‐T (PBS + 0.25% v/v Triton X‐100) for 30 min. After washing with PBS, sections were incubated with 5% v/v normal goat serum (NGS) in PBS‐T for 1 h at RT. The primary antibody (anti‐NeuN) was incubated overnight at 4 °C, diluted 1:300 in (5% v/v NGS, 0.25% v/v Triton‐X in PBS). The next day, slides were washed with PBS and incubated with the secondary antibody diluted 1:1000 in PBS for 1 h at RT. After washing, nuclei were stained with Hoechst 33258 (10 µg mL^−1^) in PBS for 10 min at RT. Sections were washed with PBS and mounted with Fluoromount G (Thermo Fisher Scientific). Images were captured using a Zeiss LSM 880 in the tile scan mode with a 10× objective. Cells were analyzed using the Cell Counter plugin in Fiji ImageJ. Antibodies are provided in Table 3 and 4.

### 2.10 Histological Analysis of the Brain and the Spinal Cord

Mice were deeply anesthetized and perfused transcardially with PBS (pH 7.4) followed by 4% w/v PFA in PBS for 10 min. After overnight postfixation, tissues were embedded in paraffine and cut into 5 µm thick sections. Paraffin sections were deparaffinized 3 times for 10 min in xylol and rehydrated in a descending ethanol series. Brain and spinal cord sections were washed for 2 min in tap water and briefly rinsed in distilled water. Sections were then incubated in Hemalum solution acid according to Mayer (Roth) for 2 min and briefly rinsed in distilled water, which was followed by a 10 min incubation in 0.1% w/v cresyl violet solution (Abcam) at 60 °C. Following a short rinse in distilled water, sections were incubated in 95% v/v ethanol for 10 min. Then, sections were dehydrated and mounted with Entellan (Merck). Images were acquired with a binocular light microscope. Lumbar spinal motoneurons (α‐motoneurons) were identified by location, size, and morphology and counted in one section per animal.

### 2.11 Skeletal Muscle and Neuromuscular Junctions

The musculus tibialis anterior was dissected from perfusion‐fixed mice. Muscles were cryo‐sectioned into 4 µm thick sections. For histological analyses, sections were stained with H&E (Sigma‐Aldrich). Images were captured with a Zeiss AxioLab A1 microscope and further analyzed with ImageJ. For immunofluorescence staining, muscle sections were permeabilized with 0.25% v/v Triton X‐100 in PBS for 10 min, blocked with 5% v/v NGS for 1 h, and incubated with primary antibodies overnight at 4 °C. After washing with PBS, sections were incubated with the corresponding secondary antibodies at a dilution of 1:1000 for 1 h at room temperature. Nuclei were stained with Hoechst 33258 (10 µg mL^−1^) (Invitrogen). Sections were washed with PBS and mounted using Fluoromount‐G (Thermo Fisher Scientific). Images were acquired using a Zeiss Cell Observer microscope. Antibodies are provided in Table 3 and 4.

For the analysis of neuromuscular junctions (NMJs), muscles were freshly dissected from 12 months old mice, fixed in 2% w/v PFA for 15 min, and subsequently washed with PBS. Fiber bundles were dissected and permeabilized with 0.2% v/v Triton X‐100 in PBS, blocked with 5% v/v NGS for 1 h, followed by an incubation with α‐Bungarotoxin‐Alexa 555 1:500 and the mouse anti‐NF200 antibody overnight at 4 °C. After washing with PBS, the myofiber bundles were incubated with the corresponding secondary antibodies at a dilution of 1:1000 for 1 h at room temperature. Nuclei were stained with Hoechst 33258 (10 µg mL^−1^). Myofibers were washed with PBS and mounted using Fluoromount. Images were captured using a Zeiss LSM 880 confocal microscope with Airyscan using the *z*‐stack module. *Z*‐projections with maximum intensities processed using ImageJ were shown.

### 2.12 Ultrastructural Analysis

For transmission electron microscopy (TEM) of tissue sections, animals were perfused with 4% w/v formaldehyde and 2.5% v/v glutaraldehyde in PBS. After dissection, tissues were postfixed overnight. 200 µm thick tissue slices were prepared on a vibratome and contrasted with 1% w/v osmium tetroxide in 100 mm sodium cacodylate buffer (Serva), dehydrated, and infiltrated with araldite resin (Agar Scientific). Ultrathin sections (60 nm thickness) were stained with uranyl acetate and lead citrate, mounted on copper grids, and viewed with a EM 900 (Zeiss, Oberkochen, Germany) transmission electron microscope. Images of cortical motoneurons were taken at 20 000× magnification and later stitched in Photoshop CS5 (Adobe). From spinal cord sections, images of three spinal motor neurons in the ventral horn of the lumbar spinal cord were acquired with 12 000× magnification with the panorama tool from ImageSP (TRS, Moorenweis, Germany). The length and number of individual ER structures were measured using FIJI ImageJ in TEM images of ultrathin sections. To avoid subjectivity, the analysis was done independently by 2 trained investigators. The mean value of all cells per animal was considered as one data point for the final evaluation. The number of individual ER structures was normalized to the size of the cytoplasm, which was assessed by the total area of the cell minus the area of the nucleus (total cell area minus the nuclear area).

### 2.13 Purification of Recombinant Proteins

TRX–His fusion proteins were purified from *E. coli* treated with 0.5 mm IPTG (overnight, 18 °C). Eluted proteins were concentrated using Amicon Ultra‐4‐10k centrifugal filter units (Millipore) and then dialyzed against liposome buffer (25 mm HEPES–KOH, pH 7.2; 25 mm KCl; 2.5 mm magnesium acetate; 100 mm potassium glutamate).

### 2.14 *REEP1* Modeling and Simulations

The human *REEP1* (UniProt accession: Q9H902‐1) was used to map the protein sequence of the WT and the exon5‐deletion variant, aligned using AlignME,^[^ ^24^, ^25^ ^]^ and visualized with Jalview.^[^ ^26^ ^]^ *REEP1* structural models (monomers and dimers) of the WT and the exon 5 deletion variant were obtained using AlphaFold2.^[^ ^27^ ^]^ These models served as initial configurations for subsequent coarse‐grained molecular dynamics simulations. The amphipathic nature of the long C‐terminal helices in the WT and exon5‐deletion variant was quantified by measuring average hydrophobicity and hydrophobic moments over a running window (11 residues long) spanning the entire length of the helical stretch using HELIQUEST.^[^ ^28^ ^]^ All‐atom models were converted to coarse‐grained (CG) representation corresponding to the MARTINI force field (v2.2),^[^ ^29^ ^]^ using the martinize.py script.^[^ ^30^ ^]^ Local helical structure of the TM and AH segments was preserved by applying restraints based on DSSP assignments.^[^ ^31^ ^]^ The C‐terminal intrinsically disordered region of *REEP1* was treated with an adapted MARTINI force field with reduced protein–protein interaction strength (*α* = 0.6).^[^ ^32^, ^33^ ^]^ The insertion depth and orientation of the proteins within the bilayer were determined using the PPM server.^[^ ^34^ ^]^ CG protein models were then embedded in the center of large square patches (40 × 40 nm^2^) containing POPC lipids (16:0–18:1 PC) spanning a periodic box (40 × 40 × 20 nm^3^) using insane.py^[^ ^35^ ^]^ and solvated with CG water containing 150 mm NaCl, following the CHARMM‐GUI protocol^[^ ^36^ ^]^ (Table 5). All systems were initially energy‐minimized using the steepest descent algorithm for 5000 steps. This was followed by an equilibration phase, during which the protein's backbone (BB) beads were position‐restrained. Equilibration was performed under *NPT* conditions, using five short simulations (10 ns each) with progressively increasing time steps (d*t* = [1,2,5,10,20] fs).^[^ ^36^ ^]^ System temperature and pressure were maintained at 1 bar and 310 K using the Berendsen thermostat and semi‐isotropic barostat^[^ ^37^, ^38^ ^]^ during equilibration. Subsequently, production runs were carried out for 10 µs for each system under *NPT* conditions (1 bar, 310 K) with a 20 fs timestep. During the production phase, the velocity‐rescale thermostat^[^ ^37^ ^]^ and the Parinello–Rahman semi‐isotropic barostat^[^ ^39^ ^]^ were used to maintain constant temperature and pressure. Long‐range electrostatic interactions were treated using reaction field with a Coulomb cutoff of 1.1 nm and a relative dielectric constant ε_rf_ of 15. Van der Waals interactions were modeled with a cutoff of 1.1 nm, using the Verlet cutoff scheme and the potential‐shift‐Verlet modifier. All CGMD simulations were conducted using GROMACS v2020.1.^[^ ^40^ ^]^

The dynamic structure of *REEP1*‐WT and the exon5‐deletion variant was compared by measuring the root mean square fluctuations of main‐chain residue positions relative to the average structure, using the gmx GROMACS tool. To explore the conformational transitions and the structural diversity of each protein system, all the protein conformations sampled during MD simulations were clustered using the Gromos method with RMSD cutoff of 0.5 nm.^[^ ^41^ ^]^ The relative depth or height of the AH segments was computed for proteins by averaging the *z*‐component of the BB beads relative to the bilayer midplane. The densities of PO_4_ groups along the *z*‐axis were used to define the midplane and relative height of the AH segments. Membrane shapes from simulations were characterized by using a modified version of MemCurv (https://github.com/bio‐phys/MemCurv), which approximated the bilayer midplane as a Monge patch *h*(*x,y*) and computed a local shape operator ***S*** (*x,y*) at any point along the *x*–*y* plane to extract the local curvature.^[^ ^12^, ^33^ ^]^ The protein center‐of‐mass (COM) positions along the *x*–*y* plane were tracked. The eigenvalues, the trace, and the determinant of the local shape operator ***S*** (*x,y*) were computed to extract the preferred mean curvature *H*(*x,y*), Gaussian *K*_G_(*x,y*), and directional curvatures, *k*_1_(*x*,*y*) and *k*_2_(*x,y*). To extract the average membrane curvature field radially around the *REEP1* proteins, all trajectory frames were first aligned such that the protein molecule was centered and oriented parallel to the *x*‐axis using the long axis of the AH to define its in‐plane orientation. By computing the curvatures using MemCurv on a 2D grid (40 × 40 nm^2^, width = 1 nm) and averaging over 100 frames spanning the last 200 ns of the representative trajectories, the local curvature fields specific to the WT and the exon5‐deletion variant were quantified.

### 2.15 Liposome Shaping Assays

Liposomes were prepared using Folch‐fraction type I lipids (Sigma‐Aldrich).^[^ ^42^ ^]^ 5 µm protein in liposome buffer was mixed with 1 mg of these liposomes and incubated for 15 min at 37 °C. Subsequently, 20 µg of proteinase K was added, and the mixture was incubated at 37 °C for additional 45 min. For freeze‐fracture, 1–2 µL of the suspension was placed between 0.1 mm copper sandwich profiles and quickly frozen in liquid ethane/propane (1:1) cooled by liquid nitrogen. The copper sandwiches were separated in a freeze‐fracture unit BAF400 (Baltec, Liechtenstein) at −150 °C. To create a replica, the surface was subsequently covered with carbon perpendicular to the sandwiches and shadowed with platinum/carbon at a 35° angle. Replicas were removed from the copper sandwich profiles through emerging in double‐distilled water and washed with Eau de Chavel prior to mounting on copper grids.^[^ ^43^ ^]^ TEM images of liposomes were acquired on an EM 902 A (Zeiss, Oberkochen, Germany) electron microscope, and diameters of around 1000 liposomes per condition were analyzed in a blinded approach using FIJI ImageJ.

### 2.16 Anti‐FLAG M2 Co‐Immunoprecipitation

HeLa‐TRex cells expressing *REEP1* WT‐FLAG or one of the variant proteins upon induction with doxycycline and the parental HeLa‐TRex empty cell line were harvested 16 h after induction following two washes in ice‐cold PBS. The cells were pelleted at 1000 *g* at 4 °C and lysed in Triton lysis buffer (50 mm Tris‐HCl, pH 7.4, 150 mm NaCl, 0.5 mm EDTA, 10 mm NEM, 1% v/v Triton X‐100) for 15 min on an overhead rotor at 4 °C. Cell debris was pelleted by centrifugation at 16 000 *g* for 20 min at 4 °C. The supernatant was loaded onto 15 µL FLAG‐M2 agarose bead slurry (Thermo Fisher), which was then equilibrated by three washes in Triton lysis buffer. Immunoprecipitation was performed by overnight incubation at 4 °C on an overhead rotor. The beads were then washed thrice using IP wash buffer (50 mm Tris‐HCl pH 8.5, 300 mm NaCl, 0.5 mm EDTA, 10 mm NEM, 1% v/v Triton X‐100), followed by three washes in Triton lysis buffer without Triton X‐100, the last wash transferring moving the pellets to a fresh Protein LoBind (Eppendorf) tube. The beads were pelleted, the supernatant removed, and the beads were frozen at −80 °C. Antibodies are provided in Table 3 and 4.

### 2.17 Co‐Immunoprecipitation

HEK‐293T cells were transfected with either *REEP1* WT‐FLAG, *REEP1* Δexon5‐FLAG, *ATL1*‐GFP, or cotransfected with *REEP1* WT‐FLAG and *ATL1*‐GFP or *REEP1* Δexon5‐FLAG and *ATL1*‐GFP. Cells were harvested 48 h after transfection and lysed with lysis buffer (20 mm Tris‐HCl, pH 8.0, 200 mm NaCl, 0.5% v/v NP‐40, and 1 mm EDTA). ChromoTek GFP‐Trap Agarose beads (Proteintech) were equilibrated with lysis buffer. Subsequently, the protein lysate was incubated with equilibrated GFP‐Trap Agarose beads for 1 h at 4 °C. The beads were collected by centrifugation at 2500 *g* for 5 min at 4 °C and washed thrice with washing buffer (20 mm Tris‐HCl, pH 7.4, 100 mm NaCl, 0.25% v/v NP‐40). Finally, the beads were resuspended in washing buffer and 6× SDS buffer (30% v/v 2‐mercaptoethanol, 40% v/v glycerol, 6% w/v SDS, Tris, pH 6.8) and boiled for 5 min at 99 °C. Protein complexes were subjected to 12% SDS‐PAGE and analyzed by immunoblot. Antibodies are provided in Table 3 and 4.

### 2.18 Mass Spectrometry – Peptide Preparation

Mass spectrometric analysis was performed on washed, detergent‐free beads, which were thawed on ice for 10 min followed by on‐bead digest. Briefly, 25 µL of SDC buffer (50 mm Tris‐HCl pH 8.5, 1 mm TCEP, 4 mm CAA, 2% w/v SDC) was added to the beads, which were then boiled for 10 min at 95 °C. After cooling to room temperature, 25 µL of 50 mm Tris‐HCl (pH 8.5) containing 500 ng of LysC and Trypsin each was added to each sample, followed by a 2 h digestion at 37 °C. The digest was stopped by the addition of 150 µL of 1% v/v trifluoroacetic acid (TFA) in isopropanol. Peptide cleanup was performed by SDB‐RPS (Sigma‐Aldrich) stage tipping, with two plugs per stage tip. Sample solutions were added to the plugs by centrifugation, followed by washes with 200 µL 1% v/v TFA in isopropanol, 0.2% v/v TFA in water, and finally, an elution in 60 µL 80% v/v acetonitrile, 1.25% v/v ammonia in water. Peptide solutions were dried in a concentrator plus vacuum centrifuge (Eppendorf) and stored at −20 °C for liquid chromatography–mass spectrometry (LC–MS) analysis.

### 2.19 Mass Spectrometry – LC–MS Analysis

Dried peptides were reconstituted in 2% v/v acetonitrile, 0.1% v/v TFA and analyzed on a QExactive HF mass spectrometer coupled to an easy nLC 1200 (Thermo Fisher Scientific) fitted with a 35 cm, 75 µm ID fused‐silica column packed in‐house with 1.9 µm C18 particles (Reprosil pur, Dr. Maisch). The column was maintained at 50 °C using an integrated column oven (Sonation). Peptides were eluted using a nonlinear gradient of 4–28% v/v acetonitrile over 45 min and directly sprayed into the mass spectrometer equipped with a nanoFlex ion source (Thermo Fisher Scientific). Full‐scan MS spectra (300–1650 *m*/*z*) were acquired in profile mode at a resolution of 60 000 at *m*/*z* 200, a maximum injection time of 20 ms, and an automatic gain control (AGC) target value of 3 × 10^6^. Up to 15 of the most intense peptides per full scan were isolated using a 1.4 Th window for fragmentation by higher energy collisional dissociation (normalized collision energy of 28). Tandem mass spectrometry (MS/MS) spectra were acquired in centroid mode with a resolution of 30 000, a maximum injection time of 45 ms, and an AGC target value of 1 × 10^5^. Single‐charged ions, ions with a charge state of more than four, and ions with unassigned charge states were not considered for fragmentation, and dynamic exclusion was set to 20 s to minimize the acquisition of fragment spectra representing already acquired precursors.

### 2.20 Mass Spectrometry – Data Processing

MS raw data were processed using MaxQuant v2.4.2.0 with default parameters, specifically including a search for diGly peptides linked to lysine to identify ubiquitinated peptides. Acquired spectra were searched against the human “one gene per sequence” database (Taxonomy ID 9606), downloaded from UniProt (11th of August, 2023) as well as a collection of common contaminants provided by MaxQuant v2.4.2.0 using the Andromeda search engine integrated into MaxQuant v2.4.2.0.^[^ ^44^, ^45^ ^]^ Identifications were filtered to obtain false discovery rates below 1% for both peptide spectrums matches (at least 7 amino acids long) and proteins using a target‐decoy strategy.^[^ ^46^ ^]^ Protein quantification and data normalization relied on the MaxLFQ algorithm implemented in MaxQuant v2.4.2.0.^[^ ^47^ ^]^ The MS proteomics data were deposited to the ProteomeXchange Consortium via the PRIDE partner repository with the dataset identifier PXD067700″.^[^ ^48^ ^]^ For protein assignment, the spectra were correlated with the UniProt human database v2019, which included a list of common contaminants. Searches were performed with tryptic specifications and default settings for mass tolerances in MS and MS/MS spectra. Carbamidomethyl cysteine, methionine oxidation, and N‐terminal acetylation were defined as fixed modifications, and diGly modification of lysine residues was described as a variable modification. The match‐between‐run feature was used with a 1 min time window. For further analysis, Perseus v2.0.3.0 was used, first filtering for contaminants and reverse entries as well as proteins that were only identified by a modified peptide. For ubiquitination pattern analysis, Perseus v2.0.3.0 was used to perform *t*‐test analysis comparing modified peptide intensities as determined by the Andromeda search engine and positive significance was assigned as given. GO term enrichment analysis was performed using the Enrichr gene list analysis tool.^[^ ^49^, ^50^, ^51^ ^]^

### 2.21 *REEP1* Degradation Assay

HeLa‐TRex cells expressing *REEP1* WT‐FLAG were seeded and induced the following day for 24 h with 1 µg mL^−1^ doxycycline. To prevent overexpression, the medium containing doxycycline was removed, the cells were washed in PBS, and fresh medium was added containing DMSO, BafA1, or MG‐132 at concentrations previously mentioned. Cells were incubated for further 24 h, after which they were harvested and lysed as described for the anti‐FLAG M2 co‐immunoprecipitation. The protein concentration was measured by BCA assay (Thermo Fisher) and samples were equalized to the same concentration within replicate groups. The supernatant was mixed with SDS running buffer to 1× concentration, followed by Western Blotting as previously described. Antibodies are provided in Table 3 and 4.

### 2.22 Statistical Analysis

Data were analyzed with Prism Version 7 and 8 (GraphPad Software, Inc.). Outliers were identified by Grubbs test. Kolmogorov–Smirnov test was used to test for normal distribution. For normally distributed data sets from more than two groups with equal variances significance was calculated by one‐way ANOVA and Tukey's post‐hoc test. Data sets from two groups at different time points were compared by two‐way ANOVA. Comparisons between normally distributed data from two groups used either two‐tailed paired or unpaired Student's *t*‐test, depending on whether the samples were related or unrelated, respectively. Not normally distributed data were tested by Mann–Whitney *U* test or Kruskal–Wallis test followed by Dunn's post‐hoc test. The respective tests used were mentioned in the figure legends along with sample sizes. Differences were considered statistically significant with *p < 0.05, **p < 0.01, ***p < 0.001, **** *p* < 0.0001. Data were presented as mean ± standard deviation (SD), if not indicated otherwise.

## 3 Results

### 3.1 KI Mice with *REEP1* Exon 5‐Deletion Display Gene‐Dosage‐Dependent Motor Deficits

**Figure 1:**
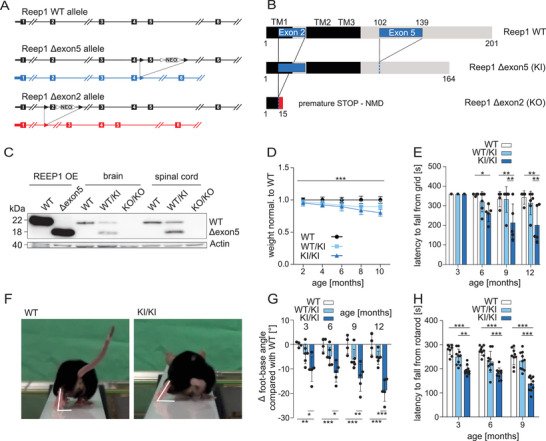
Gene‐dosage‐dependent motor deficits in mice with in‐frame deletion of *REEP1* exon 5. A) Genomic structure of the murine *REEP1* locus and the recombinant alleles with either deletion of exon 5 (in frame, KI allele) or exon 2 (out of frame, KO allele). Boxes: exons. Black triangles: loxP sites. White triangles: FRT sites; NEO: neomycin selection cassette. B) Predicted WT and recombinant allele products. The WT protein harbors 3 N‐terminal hydrophobic transmembrane (TM) regions. The TM2 and TM3 fold into a transmembrane hairpin structure, a membrane‐shaping structural motif. The C‐terminus is located on the cytoplasmic side. C) Immunoblot analysis of brain and spinal cord lysates with an antibody directed against the C‐terminal part of the *REEP1* protein. HEK‐293T cells transfected with either *REEP1* WT or the Δexon5 variant served as controls. Actin was used as a loading control. D) The body weight of heterozygous WT/KI and homozygous KI/KI mice normalized to WT decreases with age (*n* = 8 WT, 11 WT/KI, and 9 KI/KI mice; two‐way ANOVA; *** *p* < 0.001). E) From 6 months of age onward, the latency until KI/KI mice fall off an inverted grid progressively decreases (*n* = 4 WT, 6 WT/KI, and 5 KI/KI mice; two‐way ANOVA; * *p* < 0.05; ** *p* < 0.001). F,G) Compared to WT mice, the hind limb foot‐base‐angle (FBA) at toe‐off‐position progressively decreases in KI/KI mice (∆ foot‐base angle; *n* = 4 WT, 6 WT/KI, and 5 KI/KI mice; two‐way ANOVA; * *p* < 0.05; ** *p* < 0.01; *** *p* < 0.001). H) The latency to fall off an accelerating rotating rod is diminished for KI/KI mice (*n* = 4 WT, 6 WT/KI, and 5 KI/KI mice; two‐way ANOVA; ** *p* < 0.01; *** *p* < 0.001). Quantitative data are shown as mean ± SD.

The deletion of exon 2 of *REEP1* results in a frameshift mutation with a premature stop codon in exon 3 thus representing a KO allele (**Figure** **1**A,B). As reported previously, *REEP1* WT/KO and *REEP1* KO/KO mice develop a spastic gait disorder and degeneration of corticospinal axons compatible with HSP in a gene‐dosage‐dependent manner.^[^ ^23^ ^]^ To study the pathophysiology of *REEP1*‐associated dHMN, we now modeled the deletion of exon 5 in mice, which is caused by the splice‐site mutation c.304‐2A>C.^[^ ^23^ ^]^ For targeting, we inserted a loxP site into intron 4 and a Neomycin selection cassette flanked by loxP sites into intron 5. After homologous recombination in R1 ES cells,^[^ ^52^ ^]^ we transiently expressed Cre‐recombinase to delete exon 5 and the selection cassette (Figure 1A). Recombinant cells were injected into donor blastocysts and transferred into foster mice to establish the line. Western blot analysis of brain and spinal cord protein lysates from heterozygous (WT/KI) mice confirmed the presence of a truncated *REEP1* variant at the expected size (Figure 1C). WT/KI and KI/KI mice were born with an expected Mendelian ratio and exhibited a delayed gain in body weight (Figure 1D). We performed inverted screen tests to study whether WT/KI and KI/KI mice develop symptoms compatible with dHMN. Notably, the latency of KI/KI mice to fall off the screen decreased with age, compatible with an age‐dependent weakness of the forelimbs (Figure 1E). We also quantified the foot‐base angle of the hind paw at toe‐off‐position for mice walking on a beam over time (Figure 1F). A significant decrease of the foot‐base angle was first observed in 3 months old KI/KI mice, while the foot‐base angle flattened later in WT/KI mice (Figure 1G). Motor deficits were also evident in the rotarod analysis (Figure 1H). In summary, hetero‐ and homozygous KI mice develop motor impairments compatible with dHMN. Because symptoms occurred earlier and more pronounced in homozygous KI compared to heterozygous KI mice, we focused in the following sections at the comparison of KO/KO and KI/KI mice.

### 3.2 KI/KI Mice Show Degeneration of Peripheral Nerve Fibers

**Figure 2:**
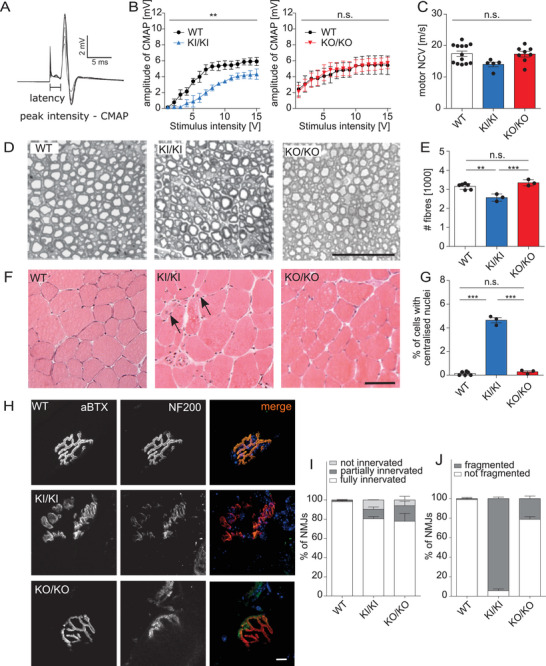
KI/KI mice but not KO/KO mice develop a progressive motor neuropathy. A–C) Amplitudes of distal compound muscle action potentials (CMAPs) upon stimulation at the tail root and motor nerve conduction velocities were not changed in KO/KO but in KI/KI mice at 6 months of age (*n* = 6 WT, 5 KI/KI, and 8 KO/KO mice; two‐way ANOVA; ** *p* < 0.01; n.s.: not significant). D) Transversal semithin sections of proximal sciatic nerves from 12 months old mice. Scale bar: 100 µm. E) Quantification of sciatic nerve fibers (*n* = 6 WT, 3 KI/KI, and 3 KO/KO mice; one‐way ANOVA with Tukey's post‐hoc test; ** *p* < 0.01; *** *p* < 0.001, n.s.: not significant). F) Representative cross sections of the musculus tibialis anterior (TA) of 12 months old mice with degenerating skeletal muscle fibers in KI/KI mice (arrows). Scale bar: 100 µm. G) Quantification of centralized nuclei in the TA in 12 months old mice (*n* = 6 WT, 3 KI/KI, and 3 KO/KO mice; one‐way ANOVA with Tukey's post‐hoc test; *** *p* < 0.001). H) Representative images of neuromuscular junctions (NMJs) from the TA at 12 months of age. Αlpha‐Bungarotoxin (αBTX, red) labels acetylcholine receptors. Neurofilament 200 (NF200, green) labels innervating nerve fibers. Scale bar: 5 µm. I,J) Quantification of not, partially, and fully innervated (I) as well as fragmented (J) NMJs (*n* = 7 WT, 3 KI/KI, and 4 KO/KO mice). Quantitative data are presented as mean ± SD, except for CMAP amplitudes, which are shown as mean ± standard error of the mean (SEM) for clarity.

As a functional readout for dHMN, we measured coCMAPs after electrical stimulation of peripheral nerve fibers. Consistent with a defect of peripheral motor nerve fibers, CMAPs were diminished in 6 months old KI/KI but not in KO/KO mice (**Figure** **2**A,B; Figure S1A,B, Supporting Information). The motor nerve conduction velocities (NCVs) (Figure 2C; Figure S1C,D, Supporting Information) and sensory amplitudes (Figure S1E,F, Supporting Information) were not changed at 6 months of age in both KI/KI and KO/KO mice. At 6 months of age, however, we observed a mild decrease in the sensory NCV in KI/KI mice (Figure S1G, Supporting Information) but not in KO/KO mice (Figure S1H, Supporting Information). Both motor and sensory NCVs were decreased in 12 and 24 months old KI/KI mice (Figure S1I–P, Supporting Information). To identify a morphological correlate for the functional deficit, we dissected proximal sciatic nerves from WT, KO/KO, and KI/KI mice at 12 months of age. We found that the number of axons was significantly decreased in KI/KI mice, while axon numbers in KO/KO mice did not differ from that of WT mice (Figure 2D,E). When we analyzed axon numbers by their diameter size, we found that mainly large‐diameter axons were lost (Figure S2A–E, Supporting Information). While the mass of the musculus tibialis anterior (TA) was not changed at 6 months of age, it was decreased in 12 months old KI/KI mice (Figure S2F,G, Supporting Information). In accordance, we found grouped degenerating skeletal muscle fibers (Figure 2F), a reduction of the mean skeletal muscle fiber diameter (Figure S2H,I, Supporting Information), more centralized nuclei (Figure 2F,G), and an increased number of CD68‐positive macrophages (Figure S2H,J, Supporting Information) in the TA of 12 months old KI/KI mice. We also stained individual TA muscle fibers with α‐bungarotoxin (αBTX), which labels acetylcholine receptors, and NF200 to label peripheral nerve endings (Figure 2H). Compared with WT samples, a considerable fraction of NMJs were only partially or not innervated in KO/KO and KI/KI samples (Figure 2I; Figure S2K,L, Supporting Information). While most NMJs were fragmented in KI/KI mice, this was rare in KO/KO and absent in WT samples (Figure 2J; Figure S2M,N, Supporting Information).

Taken together, KI/KI mice but not KO/KO mice show typical symptoms of a progressive peripheral motor neuropathy.

### 3.3 KI/KI Mice Do Not Exhibit a Pathology of Cortical Motoneurons

**Figure 3:**
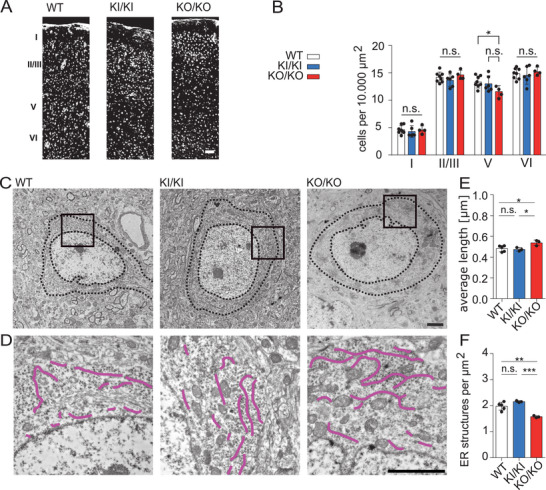
Decreased ER complexity and loss of cortical motoneurons in KO/KO but not KI/KI mice. A) Representative 10 µm sagittal cryosections of the motor cortex of 12 months old mice stained for the neuronal marker NeuN (Rbfox3). Scale bar: 200 µm. B) Quantification of neurons per 10 000 µm^2^ per layer (*n* = 8 WT, 6 KI/KI, and 4 KO/KO mice; two‐way ANOVA with Bonferroni post‐hoc test and Student's *t*‐test for the comparison of WT and KO/KO motoneurons; * *p* < 0.05). C,D) Representative transmission electron microscopy (TEM) images (magnification 3000×) of layer V motor neurons from 12 months old mice. Dashed lines mark the cell border and the nuclear membrane. Insets indicate the magnifications (magnification 20 000×) displayed in (D) with ER fragments colored in pink. Scale bars: 2 µm. E,F) Quantification of the length (E) and number (F) of individual ER structures in cortical motor neurons (≥6 cells per animal from *n* = 5 WT, 3 KI/KI, and 3 KO/KO mice). One‐way ANOVA with Tukey's post‐hoc test; * *p* < 0.05; ** *p* < 0.01; *** *p* < 0.001). Quantitative data are shown as mean ± SD.

HSP is considered a disorder of cortical motoneurons and their axons. The analysis of the motor cortex of 12 months old mice showed no change of pyramidal cells in layer V of the motor cortex of KI/KI compared to WT mice (**Figure** **3**A,B; Figure S3A, Supporting Information). By contrast, the number of motoneurons was decreased in KO/KO mice compared to WT mice at 12 months of age (Figure 3A,B; Figure S3B, Supporting Information). Motivated by our previous observation that the somatic ER was less complex in motoneurons of *REEP1* KO/KO mice,^[^ ^23^ ^]^ we compared the architecture of the ER in layer V cortical motoneurons of WT, KO/KO, and KI/KI mice by TEM (Figure 3C,D). Again, we observed an increase in the length of individual ER structures (Figure 3E) and a decrease in the number of ER structures per cell area (Figure 3F) in cortical layer V motoneurons of KO/KO mice, while the cumulative length of the ER structures did not differ between genotypes (Figure S3C, Supporting Information). We also found that the numbers of mitochondria were increased in KO/KO cortical motoneurons while their average size was decreased (Figure S3D–G, Supporting Information). Notably, we did not detect any apparent alterations in the ER or mitochondria in cortical motoneurons of KI/KI mice. Similar to cultured primary neurons from KO/KO mice,^[^ ^23^ ^]^ the axon outgrowth of cultured primary neurons isolated from KI/KI mice did not differ from those of controls (Figure S3H,I, Supporting Information).

We conclude that the homozygous deletion of *REEP1* exon 5 does not result in a significant defect of cortical motoneurons as observed in KO/KO mice.

### 3.4 ER Fragmentation Precedes Progressive Loss of Spinal Motoneurons in KI/KI Mice

**Figure 4:**
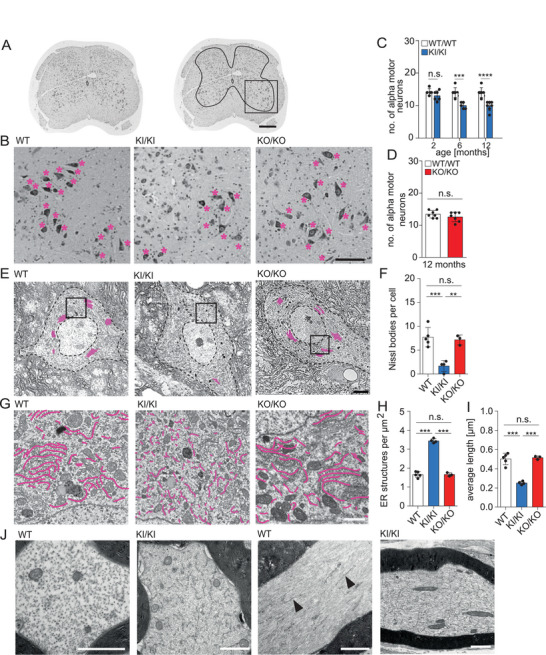
ER fragmentation and progressive loss of spinal motor neurons in KI/KI but not KO/KO mice. A) 5 µm thick Nissl‐stained cross sections of the lumbar spinal cord of 12 months old mice. In the right panel, the gray matter and the anterior horn, where α‐motoneurons are located, are marked. B) Magnifications from the ventral horn of Nissl‐stained lumbar spinal cord cross sections. Motor neurons are labelled by asterisks. Scale bar: 200 µm. C) Quantification of α‐motoneurons at indicated time points (2 months: *n* = 5 WT, 4 WT/KI, and 6 KI/KI mice; 6 months: *n* = 7 WT and 5 KI/KI mice; 12 months: *n* = 7 WT and 7 KI/KI mice; one‐way ANOVA with Tukey's post‐hoc test; * *p* < 0.05; ** *p* < 0.01; *** *p* < 0.001). D) No loss of α‐motoneurons in 12 months old KO/KO mice (*n* = 7 WT and 7 KO/KO mice; Student's unpaired *t*‐test; n.s.: not significant). E) Representative TEM images (magnification: 4000×) of 60 nm ultrathin sections of α‐motoneurons from 12 months old mice. Nissl bodies are marked in pink. Scale bar: 5 µm. F) Quantification of Nissl bodies per cell (≥3 cells per animal from *n* = 5 WT, 4 KI/KI, and 3 KO/KO mice; one‐way ANOVA with Tukey's post‐hoc test; ** *p* < 0.01; *** *p* < 0.001). G) Representative TEM images (magnification 12 000×) from the cytoplasm of lumbar spinal motoneurons from 12 months old mice. In the lower panel, individual ER structures are marked in pink. Scale bar: 1 µm. H,I) Quantification of the number (H) and length (I) of individual ER structures in α‐motoneurons (≥3 cells per animal from *n* = 5 WT, 4 KI/KI, and 3 KO/KO mice; one‐way ANOVA with Tukey's post‐hoc test; *** *p* < 0.001). J) TEM images of cross‐ and longitudinal sciatic nerve sections at 12 months of age. Arrowheads indicate the typical ER morphology in WT samples. In KI/KI mice, we found focal accumulations of individual ER fragments often showing a ladder‐like appearance in longitudinal sections. Scale bars: 1 µm. Quantitative data are shown as mean ± SD.

Given the loss of peripheral nerve fibers and grouped muscular atrophy, we quantified α‐motoneurons in 12 months old WT, KO/KO, and KI/KI mice. Spinal motoneurons were identified by their localization in the ventral horn as well as their large size and typical morphology (**Figure** **4**A,B). Consistent with a loss of axons in sciatic nerves of KI/KI mice, we found a loss of α‐motoneurons in 6 and 12 months old KI/KI (Figure 4C), but not in KO/KO mice (Figure 4D). Therefore, we studied the ultrastructure of spinal motoneurons. In WT and KO/KO samples, we observed the typical stacks of ER sheets also known as Nissl‐bodies, which were small and reduced in number in spinal motoneurons of KI/KI mice (Figure 4E,F; Figure S4A–D, Supporting Information). Overall, the number of individual ER fragments was drastically higher in KI/KI mice, while their average length was decreased (Figure 4G–I). This was also evident when we excluded Nissl bodies from the analysis (Figure S4E,F, Supporting Information). The cumulative length of ER structures in α‐motoneurons did not differ between genotypes (Figure S4G, Supporting Information). Notably, the morphological features of the ER in KO/KO spinal motoneurons were not distinguishable from WT. We also assessed the morphology of the ER in proximal sciatic nerve axons of 12 months old KI/KI mice (Figure 4J) and found a fragmentation of the ER as well as ladder‐like expansions of transverse ER‐sheet structures. The latter were also reported for *ATL1* and *REEP1* double mutant mice^[^ ^53^ ^]^ and for *Arl6ip1* KO mice.^[^ ^54^ ^]^

Thus, KI/KI but not KO/KO mice exhibit an age‐dependent loss of spinal motoneurons and a more fragmented ER structure in spinal motoneurons.

### 3.5 The ΔExon5 Variant Retains the Membrane‐Shaping Properties of *REEP1*

**Figure 5:**
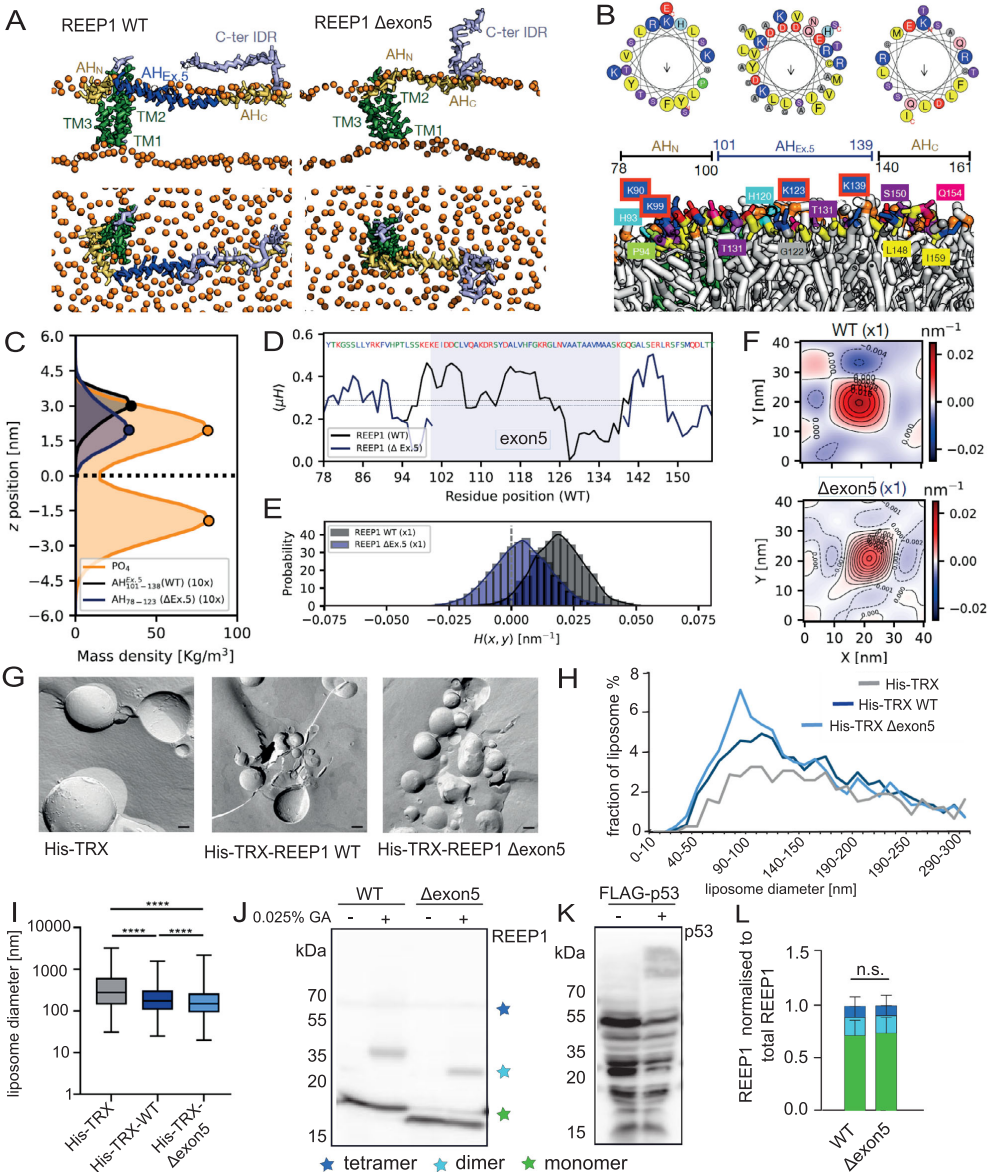
Deletion of exon5 largely preserves the membrane remodeling properties of *REEP1*. A) Representative snapshots (top and side views) of *REEP1* WT (left) and *REEP1* Δexon5 (right) from 10 µs long MD simulations. The three TM segments (green) and the AH segments (yellow), along with the exon5 region (blue), anchor the protein in the POPC bilayer (PO_4_ groups in orange). Note the shorter footprint of *REEP1* Δexon5. B) The AH of WT *REEP1* partitions at the bilayer/water interface with the hydrophobic side (yellow labels) embedded in the upper leaflet (head groups orange, tails in white) and the polar side facing the cytosol (colored labels). The long AH can be divided into an N‐terminal AH_N_ kinked at P94, a central AH_Ex.5_, and the C‐terminal AH_C_. Possibly ubiquitinated lysine residues are highlighted with a red outline. C) Density profiles of AH segments of WT (black) and *REEP1* Δexon5 (blue) variants (magnified ×10 for clarity) along the *z*‐axis. Profiles are shown relative to the midplane (dotted line), with headgroup positions marked on either side (orange). D) Hydrophobic moment ⟨*µH*⟩ along the length for the WT AH segment (⟨*µH*⟩ = 0.28 ± 0.11) and the *REEP1* Δexon5 AH segment (⟨*µH*⟩ = 0.26 ± 0.11), indicating a slight drop. E) Probability densities (and histograms) of intrinsic mean curvature sampled by the WT (*H*(*x,y*) = 0.018 nm^−1^) and *REEP1* Δexon5 (*H*(*x,y*) = 0.004 nm^−1^). F) Protein‐induced mean curvature fields illustrating local membrane perturbation due to monomeric WT (top) and *REEP1* Δexon5 (bottom). G) Freeze‐fracture TEM images of liposomes after incubation with the indicated recombinant proteins. Scale bar: 200 nm. H) Liposome diameters after incubation with His–TRX, His–TRX–*REEP1* WT, and His–TRX–*REEP1* Δexon5 recombinant proteins. I) Box plots of the complete data set are partially presented in (H). Boxes contain 50% of the values; minimal, maximal, and median ($\tilde{x}$) values are marked by vertical lines. Note the logarithmic *y*‐axis (*n* = 2 independent experiments; Kruskal–Wallis test with Dunn's post‐hoc test **** *p* < 0.0001). J–L) *REEP1* Δexon5 still homodimerizes. (I) Representative immunoblot of *REEP1* WT and Δexon5 following chemical cross‐linking with glutaraldehyde (GA). Green, light blue, and dark blue stars indicate the size of the monomer, dimer, and higher‐order complexes, respectively. (K) FLAG‐p53 served as a positive control. (L) Quantification of *REEP1* monomers, dimers, and tetramers (*n* = 3 experiments; two‐way ANOVA). Quantitative data are shown as mean ± SD.

To study the structure and functional properties of *REEP1* WT and the exon5‐deletion (Δexon5) variant, we modeled their monomeric and dimeric structures and performed coarse‐grained molecular dynamics simulations in model lipid bilayers (**Figure** **5**A; Figure S5A, Supporting Information). We found that the WT and the variant protein remained stable in POPC bilayers. The TM segments compressed the bilayer locally and adjusted their relative depth, relaxing to a more stable conformation (Cluster 1 in Figure S5E in the Supporting Information). The WT protein had a larger membrane footprint due to its long C‐terminal AH (83 residues). We could further see that the AH could be partitioned into three segments, an AH_N_ flanking the cytosolic end of TM3, followed by the AH_Ex.5_ corresponding with the region encoded by exon 5, and the C‐terminal AH_C_ (Figure 5B). By contrast, the *REEP1* Δexon5 has a substantially shorter AH segment (Figure S5B, Supporting Information, 43 residues, AH_N_ fused with AH_C_). Analysis of the helix properties revealed that the AH of *REEP1* Δexon5 displayed a marginally reduced mean hydrophobic moment and was buried slightly below the phosphate layer of the cytosolic leaflet (Figure 5B–D; Figure S5C, Supporting Information). Dimeric *REEP1* WT and Δexon5 displayed a much larger footprint in the bilayer, locally deforming it (Figure S5A, Supporting Information). Fluctuation analysis showed that the TM1, TM2, and the intervening cytosolic segments stabilized the dimer configuration and the antiparallel arrangement of the cytosolic AH segments (Figure S5D–F, Supporting Information). To quantify the curvature induction capacity of the WT and the exon5‐deletion variant, we tracked the COM of the proteins in the POPC bilayers and measured their induced curvature. We found that both *REEP1* WT and Δexon5 preferred regions with positive mean curvature (Figure 5E; Figure S5G, Supporting Information; *H*(*x,y*) > 0 nm^−1^). The WT monomeric protein preferred slightly higher directional curvatures (*k*_1_ = 0.02 ± 0.01 nm^−1^ and *k*_2_ = 0.009 ± 0.01 nm^−1^) as opposed to the Δexon5 monomer (*k*_1_ = 0.009 ± 0.01 nm^−1^ and *k*_2_ = −0.005 ± 0.01 nm^−1^; Figure S5H, Supporting Information, left). This pattern was also consistent with the directional curvature preference of the dimers (Figure S5H, Supporting Information, right). To quantify the local membrane deformation of the flat bilayer around the protein, we computed the local membrane curvature field radially around the protein and averaged it. In our simulations, the WT protein induced a larger and more isotropic curvature field (Figure 5F; Figure S5I, Supporting Information, top), whereas Δexon5 resulted in a smaller anisotropic curvature field (Figure 5F; Figure S5I, Supporting Information, bottom).

We tested the membrane‐shaping properties of the recombinantly expressed proteins experimentally in liposome‐shaping assays. N‐terminally His–TRX‐tagged *REEP1* WT, the Δexon5 (His–TRX–*REEP1* Δexon5), or His–TRX alone were incubated with liposomes. Liposomes were then snap‐frozen and freeze‐fractured to determine their size (Figure 5G). As expected, the fraction of small‐diameter proteoliposomes increased in the presence of His–TRX *REEP1* WT (Figure 5H,I).^[^ ^23^ ^]^ Also Δexon5 gave rise to smaller liposomes. The fraction of particularly small liposomes was even slightly increased for Δexon5 leading to a marginally decreased mean liposome diameter (Figure 5I). As the ability of *REEP1* to form dimers is essential for its membrane shaping abilities,^[^ ^13^ ^]^ we transfected HEK‐293T cells with constructs for either *REEP1* WT or Δexon5 and cross‐linked proteins by adding 0.025% glutaraldehyde. The subsequent SDS‐PAGE showed that the ability to form dimers is not compromised for Δexon5 (Figure 5J–L).

Thus, simulations showed that Δexon5 induces local membrane perturbation, generating positive membrane curvature, thereby remodeling flat bilayers consistent with the shredding of larger liposomes into smaller vesicles in vitro.

### 3.6 The ΔExon5 Variant Accumulates in KI/KI Mice

**Figure 6:**
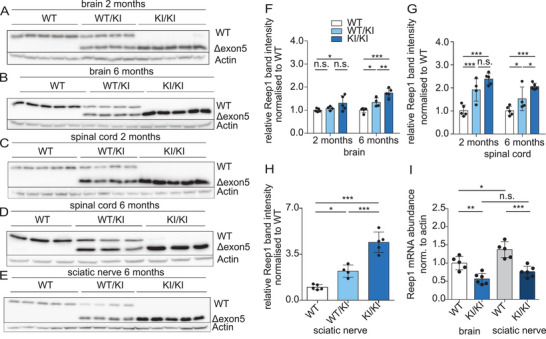
Increased abundance of the *REEP1* exon5‐deletion variant in KI/KI mice. A–E) Representative *REEP1* immunoblots of total brain, spinal cord, and sciatic nerve lysates from 2 and 6 months old mice. F) Quantification normalized to actin and WT for brain (*n* = 5 WT, 4 WT/KI, and 5 KI/KI mice; one‐way ANOVA with Tukey's post‐hoc test; * *p* < 0.05; ** *p* < 0.01; *** *p* < 0.001; n.s.: not significant). G) Quantification normalized to actin and WT for spinal cord (*n* = 5 WT, 4 WT/KI, and 5 KI/KI mice; * *p* < 0.05; *** *p* < 0.001; n.s.: not significant). H) Quantification normalized to actin and WT for sciatic nerves (*n* = 5 WT, 4 WT/KI, and 5 KI/KI mice; * *p* < 0.05; *** *p* < 0.001; n.s.: not significant). I) Relative *REEP1* transcript abundance in brain normalized to Actin and WT assessed by real‐time PCR (brain: *n* = 5 WT and 6 KI/KI mice; sciatic nerve: *n* = 5 WT, and 6 KI/KI mice; one‐way ANOVA with Tukey's post‐hoc test; * *p* < 0.05; ** *p* < 0.01; *** *p* < 0.001). Quantitative data are shown as mean ± SD.

Western blot analysis of tissue lysates obtained from 2 or 6 months old mice revealed an increase in the amount of the Δexon5 variant in KI/KI mice as opposed to *REEP1* WT levels in control mice (**Figure** **6**A–H). The increase was most prominent (roughly fourfold) in lysates of sciatic nerves, which comprise motor fibers for the hind limb.

In spinal cord lysates, the abundances of *REEP2* and *REEP3* did not differ between WT and KI/KI mice, while *REEP4* levels were increased in samples from KI/KI mice (Figure S6A,B, Supporting Information). In brain lysates from KO/KO mice, we noted an increase in *REEP3* levels but no change in *REEP2* or *REEP4* abundances compared to WT (Figure S6C,D, Supporting Information).

We next assessed whether the prominent increase of the variant *REEP1* protein is reflected by an increase in *REEP1* gene transcription. We quantified the abundance of *REEP1* mRNA transcripts isolated from brains or sciatic nerves obtained from 6 months old WT and KI/KI mice. Interestingly, the amount of *REEP1* transcripts was reduced by roughly 50% in KI/KI samples in comparison to WT, thus ruling out an upregulation of *REEP1* transcription in KI/KI mice (Figure 6I).

The increased abundance of Δexon5, despite its downregulation at the transcriptional level, suggested that the turnover of *REEP1* Δexon5 might be compromised.

### 3.7 Reduced Ubiquitination of *REEP1* ΔExon5 Is Associated with dHMN

**Figure 7:**
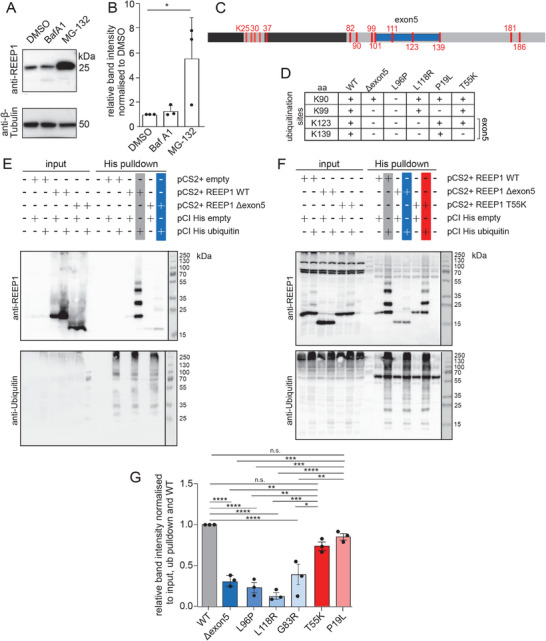
Decreased ubiquitination of *REEP1* variants associated with dHMN. A,B) *REEP1* is degraded via the proteasomal system and not by autophagy. Quantification of *REEP1* WT in stably transfected HeLa‐TRex cells upon either inhibition of the proteasome with MG‐132 or inhibition of autophagy with Bafilomycin A1 (one‐way ANOVA with Tukey's post‐hoc test; * *p* < 0.05). The same *REEP1* WT data are shown in comparison with data for selected *REEP1* variants in Figure S7A,B (Supporting Information). C) Lysine residues of *REEP1*. The transmembrane segments are depicted in dark gray and the C‐terminal part encoded by exon5 in blue. D) Ubiquitination sites identified by mass spectrometry upon induction of WT and variant *REEP1* in HeLa TRex cells. While K123 is ubiquitinated in the WT and HSP‐associated variants (P19L, T55K), dHMN‐associated variants (L96P, L118R) lack ubiquitinated K123. E–G) In vitro ubiquitination assays confirm that *REEP1* variants associated with dHMN but not with HSP are less ubiquitinated. After transfection of HEK‐293T cells with constructs encoding either the *REEP1* WT or disease‐associated variants, together with His–ubiquitin, the cells were lysed, and His–ubiquitin was pulled down with Ni‐NTA beads. Samples were analyzed by immunoblot for *REEP1* and Ubiquitin. The *REEP1* pull‐down was normalized to the *REEP1* input and the ubiquitin pull‐down. (E) Representative immunoblots for *REEP1* WT and Δexon5. The separated lanes at the right margin depict the size standard. (F) Representative immunoblots for *REEP1* WT, Δexon5, and the HSP‐associated T55K variant. The separated lanes at the right margin depict the size standard. (G) Quantification for the dHMN‐associated variants Δexon5, L96P, L118R, and G83R and the HSP‐associated variants T55K and P19L (*n* = 3 experiments; paired Student's *t*‐test; ** *p* < 0.01). Quantitative data are shown as mean ± SD.

To assess whether *REEP1* is degraded via autophagy or the ubiquitin–proteasome system, we induced the expression of FLAG‐tagged *REEP1* in HeLa‐TRex cells and either blocked autophagy with Bafilomycin A or proteasomal degradation with MG‐132. Importantly, *REEP1* accumulated upon proteasomal inhibition but not upon inhibition of autophagy (**Figure** **7**A,B; Figure S7A,B, Supporting Information), indicating that the proteasome is primarily responsible for its turnover. Because proteasomal degradation of proteins is preceded by their ubiquitination, we next studied potential ubiquitination sites of *REEP1*. We found a substantial proportion of lysine residues mapped to the C‐terminus, many of which were located in the region encoded by exon 5 (Figure 7C). We induced the expression of either the FLAG‐tagged *REEP1* WT or Δexon5 protein in HeLa‐TRex cells and performed pull‐down via the FLAG‐tag and used mass spectrometry to map the actual ubiquitination sites. We found that the WT protein was ubiquitinated at K90, K99, K123, and K139, whereas only K90 was ubiquitinated in Δexon5 (Figure 7D; Figure S7C, Supporting Information).

To assess whether the compromised ubiquitination is a consistent feature of *REEP1* variants associated with dHMN, we searched for other *REEP1* variants associated with dHMN. From the literature, we selected the L96P variant^[^ ^55^ ^]^ and a large family with dHMN.^[^ ^56^ ^]^ In the latter, we identified the novel *REEP1* variant G83R segregating with the disease. In another sporadic patient with severe axonal dHMN, we found the novel L118R variant. All dHMN‐associated variants studied appear to be targeted to the proteasome for degradation, because they accumulated in cells upon proteasomal inhibition with MG‐132 (Figure S7A,B, Supporting Information). Notably, the missense variant L96P had no ubiquitinated lysine residue, whereas in the L118R variant only positions K90 and K99 were ubiquitinated. All variants associated with dHMN lacked ubiquitination at K123 and K139 (Figure 7D; Figure S7C, Supporting Information). By contrast, *REEP1* ubiquitination was preserved in the missense variants P19L and T55K, which are associated with pure HSP.^[^ ^57^, ^58^ ^]^ To confirm these findings, we cotransfected HEK‐293T cells with constructs either encoding the WT or the variant *REEP1* protein together with His_6_–ubiquitin. After cell lysis, His_6_–ubiquitin was pulled down using Ni‐NTA beads and samples analyzed by *REEP1* immunoblot. Consistent with our mass spectrometry data, *REEP1* WT and the HSP‐associated variants P19L and T55K were significantly enriched in the ubiquitin‐pull‐down, while this was not the case for Δexon5 or the other variants associated with dHMN (Figure 7E–G), suggesting that dHMN related variants showed reduced ubiquitination.

Taken together, *REEP1* variants associated with dHMN exhibit compromised ubiquitination, leading to their accumulation and an overload of ER membranes.

### 3.8 *REEP1* Is Ubiquitinated by *HUWE1*

**Figure 8:**
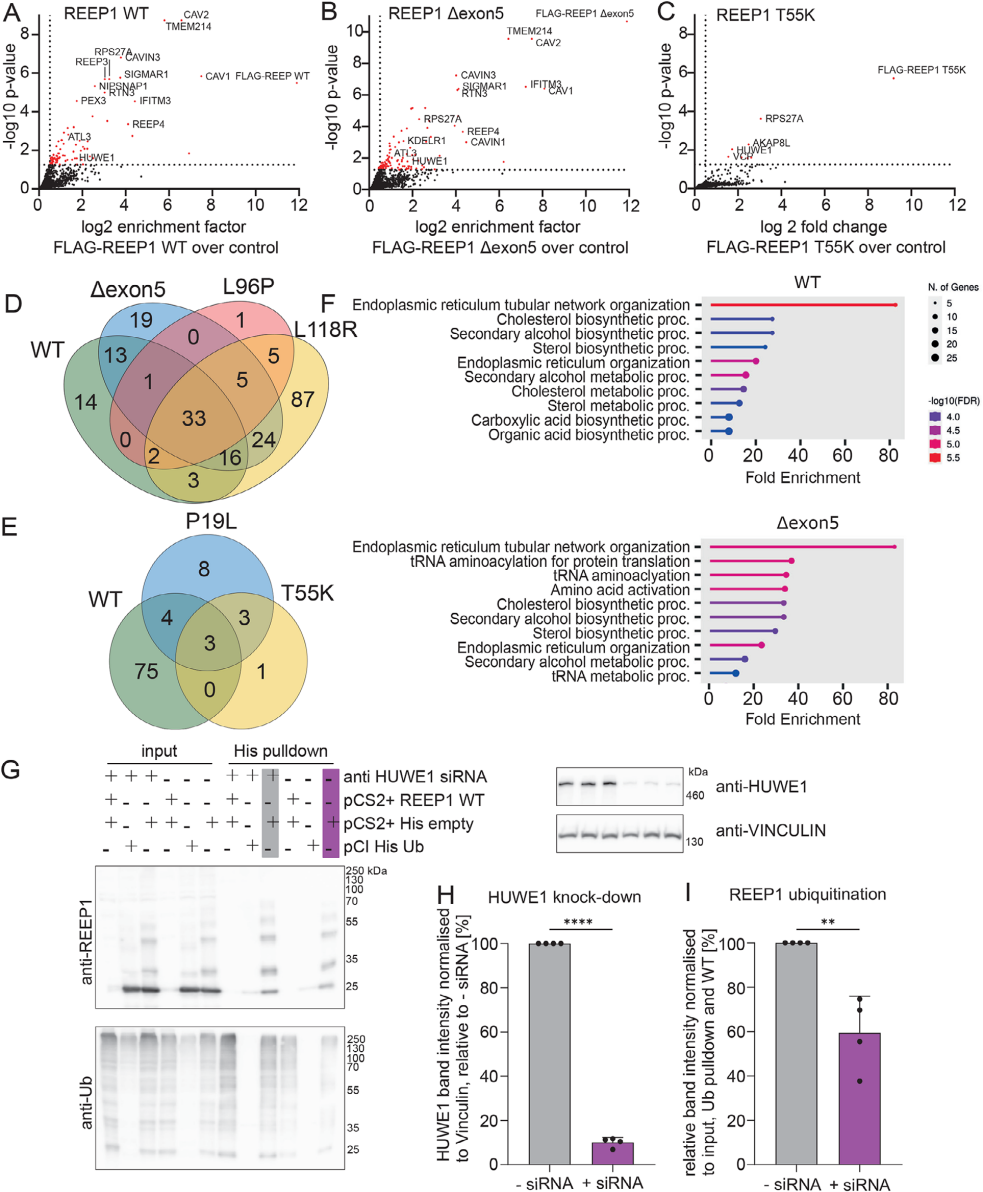
*REEP1* is ubiquitinated by *HUWE1*. A–C) Mass spectrometry HeLa TRex cells induced with doxycycline expressing *REEP1* WT‐FLAG (A), *REEP1* delta Exon5‐FLAG (B) or *REEP1* T55K‐FLAG (C) after co‐immunoprecipitation with antiFLAG‐M2 antibody compared to uninduced HeLa TRex cells (*n* = 3 experiments each, significance cutoff −log10 *p*‐value of 1.25, log2 fold change of 0.5). D) Venn diagram illustrating the overlaps between the interactomes for *REEP1* WT and dHMN‐associated variants. E) Venn diagram illustrating the overlaps between the interactomes for *REEP1* WT and HSP‐associated variants. F) GO biological process term analysis for interactomes of *REEP1* WT and the delta exon5‐deletion variant. G–I) Knockdown of *HUWE1* reduces the ubiquitination of *REEP1* in vitro. (G) Representative immunoblots of *REEP1* ubiquitination with and without siRNA‐mediated knock‐down of *HUWE1*. (H, I) Quantification of *HUWE1* knockdown (H) and *REEP1* ubiquitination (I) (*n* = 4 experiments; Student's *t*‐test; ** *p* < 0.01; **** *p* < 0.0001). Quantitative data are shown as mean ± SD.

We studied the interactomes of the FLAG‐tagged WT or variant proteins associated with dHMN or HSP upon induction of the respective proteins in HeLa‐TRex cells (**Figure** **8**A–F; Figure S7B, Supporting Information). While the interactome of the Δexon5, the L96P, and the L118R variants largely overlapped with the interactome of the *REEP1* WT protein, the HSP variants *REEP1* P19L and T55K interacted with only a few proteins, including VCP, an AAA‐ATPase associated with the ERAD pathway (Figure 8A–E; Figure S7D–F, Supporting Information).^[^ ^59^ ^]^ GO enrichment analysis indicated “endoplasmic reticulum tubular network” as the most prominent term associated with both WT and Δexon5 interacting proteins (Figure 8F).

Because *REEP1* is known to interact with the ER protein *ATL1*,^[^ ^10^ ^]^ we assessed whether this interaction might be compromised for the Δexon5 variant. Both *REEP1* WT protein and the Δexon5 variant coprecipitated with *ATL1*, suggesting that the region encoded by exon5 is apparently not essential for the interaction between both proteins (Figure S7G, Supporting Information).

As the only REEP homolog in fission yeast was shown to play a role in autophagy,^[^ ^17^, ^18^, ^19^ ^]^ we also assessed autophagy‐related proteins within these interactomes. However, we did not find any significant enrichment of autophagic proteins in the interactome, further confirming that *REEP1* is not degraded by autophagy. In agreement, the number of autophagosomes and autolysosomes upon starvation and induction of autophagy did not differ between primary neurons from WT or KO/KO mice (Figure S8A,B, Supporting Information). Neither the quantification of lipofuscin particles in cortical motoneurons by TEM (Figure S8C,D, Supporting Information), nor the immunoblot analysis for p62 or LC3B‐II in brain and spinal cord lysates from KI/KI and KO/KO mice (Figure S8E–H, Supporting Information) suggested that autophagy might be compromised. Within the interactome of *REEP1*, we identified the E3‐Ubiquitin Ligase *HUWE1* as a recurrent hit that could potentially mediate the ubiquitination of *REEP1*. The siRNA‐mediated knockdown of *HUWE1* significantly decreased the ubiquitination of *REEP1* upon cotransfection of HEK‐293T cells with constructs for *REEP1* and His_6_–ubiquitin (Figure 8G–I), thus confirming that *HUWE1* directly contributes to the ubiquitination of *REEP1* and its proteasomal targeting and degradation.

## 4 Discussion and Conclusion

HSP‐associated *REEP1* variants include larger deletions and nonsense mutations and only a few missense mutations, which likely compromise the topology of RHDs, suggesting that haploinsufficiency due to *REEP1* loss‐of‐function causes SPG31. In agreement, *REEP1* KO mice develop a gene‐dosage‐dependent phenotype consistent with HSP.^[^ ^14^, ^23^ ^]^ We previously reported a family with dHMN segregating with the *REEP1* splice site mutation c.304‐2A>C, which results in the in‐frame deletion of exon 5. Here, we report two novel variants associated with dHMN: the G83R variant, which segregates in a large family reported previously,^[^ ^56^ ^]^ and the L118R variant in a sporadic patient with severe dHMN. Remarkably, patients with the *REEP1* variant L96P were reported to result in an overlapping phenotype consistent with both HSP and dHMN.^[^ ^55^ ^]^ Overall, *REEP1* variants associated with dHMN thus appear to be restricted to the C‐terminal cytoplasmic part of the protein, but not its TM segments.

To gain insights into the respective pathophysiology of *REEP1*‐associated dHMN, we modeled the exon 5‐deletion in mice. Our KI/KI mice exhibited typical features of dHMN, including progressive loss of peripheral motoric nerve fibers and muscular atrophy in a gene‐dosage‐dependent manner, suggesting that these mice are a valid model for *REEP1*‐associated dHMN. This allowed us to directly compare the consequences for the somatic ER structure of cortical and spinal motoneurons in both KO/KO and KI/KI mice. While we confirmed a less complex ER morphology in the soma of cortical motoneurons in KO/KO mice as reported previously,^[^ ^23^ ^]^ we found a more fragmented ER in spinal motoneurons of KI/KI mice as quantified from segmentation by visual inspection of individual TEM sections. To minimize artifacts, only clear, isolated discontinuities without evidence of extension into neighboring sections were classified as distinct ER fragments. In the absence of 3D reconstructions, however, we cannot exclude the possibility of apparent fragmentation reflecting more tortuous or highly curved ER tubules. Both ER alterations could compromise critical ER functions such as protein translation, lipid synthesis, protein glycosylation, the unfolded protein response, or Ca^2+^ homeostasis, and thus explain the progressive loss of either spinal or cortical motoneurons.

*REEP1* is presumed to induce high membrane curvature by the formation of splayed dimers held together by TM segments, in combination with the shallow hydrophobic insertions of the C‐terminal amphipathic helix in the cytoplasmic leaflet of the ER membrane.^[^ ^13^ ^]^ As the exon 5 encodes a substantial part of the amphipathic helix, we speculated that the Δexon5 variant might affect the formation of homodimers, consequently affecting the membrane‐shaping properties of variant *REEP1*. Consistent with previous reports,^[^ ^13^ ^]^ our cross‐linking studies did not suggest a significant effect on the dimerization of the Δexon5 variant. Furthermore, computer modeling and simulations demonstrated that both the monomeric and dimeric versions of the wild‐type protein and Δexon5 retained the capacity to perturb the bilayer structure locally, inducing positive mean curvature. Due to the smaller amphipathic helix, the Δexon5 variant had a smaller asymmetric membrane footprint compared to the WT protein, thus resulting in a smaller anisotropic curvature field. A previous study reported for the yeast protein *YOP1p* and for the Xenopus protein *REEP5* a decrease in the formation of small lipoprotein particles (average diameter <12 nm) upon deletion of the C‐terminal amphipathic helix.^[^ ^13^ ^]^ Such small lipoprotein particles cannot represent vesicles with membrane bilayers and thus would not fracture in membrane freeze‐fraction/TEM analyses. Under in vitro conditions, both the *REEP1* WT and Δexon5 protein displayed membrane remodeling capacity. Incubation of isolated proteins with large liposomes resulted in the formation of numerous smaller vesicles. However, *REEP1* Δexon5 showed marginally enhanced membrane‐shaping properties, as observed by the slightly lower shift in the distributions of median liposome diameters. Based on our structural modeling and simulations, we hypothesize that the smaller footprint of the shorter amphipathic helix of Δexon5 allows for denser packing under saturating conditions in comparison to the WT protein. Consistent with this assumption, we found a significantly enhanced amount of mutant *REEP1* protein in brain, spinal cord, and sciatic nerve tissue lysates of KI/KI mice compared to the WT protein in WT mice, which was most prominent for sciatic nerve lysates, which include the motor fibers originating from spinal motoneurons. As a consequence, the accumulation of excess *REEP1* Δexon5 could lead to denser protein clusters, resulting in highly deformed membrane geometries and a fragmented ER structure.

As the transcript levels were significantly reduced in nervous tissues of KI/KI mice, we posit that a defective turnover and protein degradation process is responsible for the accumulation of excess protein in ER membranes of spinal motoneurons, as evidenced by Western blot analyses. In line with this, we demonstrated that the clearance of *REEP1* is primarily mediated via the proteasome, as indicated by the accumulation of WT *REEP1* upon proteasomal inhibition. Proteins destined for proteasomal turnover are tagged by ubiquitin moieties on specific lysine residues. Therefore, we assessed the ubiquitination status of induced *REEP1* after pull‐down of FLAG‐tagged *REEP1* by mass spectrometry. In the WT protein, the C‐terminal lysine residues K123 and K139 located in the region encoded by exon 5, were significantly ubiquitinated in addition to K90 and K99. The compromised ubiquitination status of *REEP1* Δexon5 can impair proteasomal degradation, resulting in its accumulation and, consequently, fragmentation of the ER due to protein overload. To determine if this is a general feature of *REEP1* variants associated with dHMN, we sought to identify additional families with dHMN linked to *REEP1* variants. Notably, all the variants related to dHMN are located in the C‐terminal part of *REEP1* and displayed a significantly diminished ubiquitination in vitro. By contrast, the ubiquitination of two missense variants associated with HSP was largely preserved. Notably, we also found an increased abundance of *REEP4* in lysates from KI/KI mice. Possibly, *REEP4* is stabilized in KI/KI mice because it forms heteromers with the Δexon5 variant, which may further increase membrane curvature.

Studying the interactome of *REEP1* enabled us to identify *HUWE1* as a cognate E3‐Ubiquitin Ligase involved in the regulation of *REEP1* turnover. Notably, we found reports that members of a large pedigree with severe syndromic mental retardation due to mutations in *HUWE1* also developed distal muscular atrophy thus reproducing the main symptom of our dHMN patients.^[^ ^60^, ^61^ ^]^ Although the HSP‐associated *REEP1* variants lost most protein interaction partners, they retained interactions with *HUWE1* in addition to gaining significant interaction with VCP, which is known to extrude ubiquitinated proteins from the ER, targeting them to cytosolic degradation.^[^ ^59^ ^]^ Therefore, we surmise that in stark contrast to the accumulation of mutant *REEP1* in dHMN variants, the HSP‐associated variants, when expressed, are ubiquitinated and preferentially targeted for proteasomal degradation, thus being unable to support *REEP1*‐mediated membrane shaping in cortical neurons.

There is still no consensus regarding the precise subcellular localization of *REEP1* or its orthologs *REEP2*, *REEP3*, and *REEP4*. Several reports localize *REEP1* to the plasma membrane,^[^ ^62^ ^]^ mitochondria,^[^ ^8^ ^]^ the ER,^[^ ^10^ ^]^ or more recently ER‐related novel vesicular compartments of unknown function.^[^ ^20^ ^]^ These conflicting findings are likely owed to the lack of antibodies to detect endogenous *REEP1* in brain tissue sections. We previously reported that *REEP1* detection overlaps with the tubular ER marker *RTN4* in subcellular fractions of brain lysates.^[^ ^23^ ^]^ Moreover, heterologously expressed *REEP1* was localized to ER tubules and increased the alignment of ER tubules along microtubules in COS7 cells.^[^ ^10^ ^]^ Although reports exist on the involvement of fission yeast homologue of *REEP1* in autophagosome formation,^[^ ^17^, ^18^, ^19^ ^]^ we did not observe an accumulation of *REEP1* upon inhibition of autophagy with Bafilomycin. In addition, there was no accumulation of LC3‐II and p62/SQSTM1 in protein lysates of KO/KO mice, thus excluding a major involvement of *REEP1* in autophagy. Moreover, the quantification of autophagosomes and autolysosomes in primary motoneurons isolated from WT and KO/KO mice yielded comparable numbers both at steady state and after induction of autophagy by Torin1 and EBSS starvation consistent with a normal autophagic flux in the absence of *REEP1*. Further supporting the notion that mammalian *REEP1* is not directly linked with autophagy, we identified only very few autophagy‐related proteins in the *REEP1* interactome. These were mostly ER‐localized ER‐phagy receptors such as *RTN3*,^[^ ^63^ ^]^ which also possess RHDs and may display homotypic interactions with REEPs without initiating autophagy. Instead, our mass‐spectrometry‐based analysis of the interactome of WT and the *REEP1* Δexon5 yielded proteins related to the organization of the ER tubular network as top hits. In agreement, the deletion of the yeast orthologue *YOP1p* caused a loss of ER tubules,^[^ ^11^ ^]^ which may correlate with our findings in cortical motoneurons of KO/KO mice. The overexpression, however, led to the formation of bundles of ER tubules,^[^ ^64^, ^65^ ^]^ which may correspond with our findings in spinal motoneurons and sciatic nerve fibers of KI/KI mice.

Interestingly, SaOS2 and Bewo cells express *REEP1* at high levels,^[^ ^66^ ^]^ thus allowing the localization of endogenous *REEP1* together with *ATL1* to vesicles and not to ER tubules.^[^ ^20^ ^]^ While the function of these vesicles is unclear, it was speculated that the budding and fusion of these vesicles may allow ER tubules to shrink and grow, thus cooperating with Atlastins to generate dynamic tubules.^[^ ^20^ ^]^ The localization of *REEP1* to an ER‐related vesicular compartment is not in conflict with our findings, as it has been shown that several disease‐associated *REEP1* variants including Δexon5 were retained in the ER in these cells, which may cause the ER overload and subsequent fragmentation of the ER in KI/KI mice. The GTPase *ATL1* plays an essential role in the formation of three‐way junctions in the ER and interacts with *REEP1*.^[^ ^21^, ^22^ ^]^ Notably, *ATL1* variants are also associated with HSP.^[^ ^67^, ^68^, ^69^ ^]^ Accordingly, it was proposed that *REEP1* and *ATL1* coordinate dynamic ER shaping and microtubule reorganization in corticospinal neurons.^[^ ^10^ ^]^ Of note, the combined inactivation of *ATL1* together with the KO of *REEP1* led to pronounced abnormalities of the ER in corticospinal tract fibers with ladder‐like structures,^[^ ^53^ ^]^ similar to our findings in sciatic nerve fibers of KI/KI mice.^[^ ^48^ ^]^

Surprisingly, the accumulation of the Δexon5 variant was much more pronounced in the sciatic nerve compared to brain lysates, and the ER fragmentation was only observed in spinal but not cortical motoneurons of KI/KI mice. By contrast, the KO of *REEP1* only affected the ER of cortical but not spinal motoneurons. The abundance of the membrane shaping protein *REEP1* in the ER must be tightly regulated to meet the different requirements in cortical and spinal motoneurons. Since the siRNA‐mediated knockdown of *HUWE1* did not altogether abolish ubiquitination of *REEP1*, additional E3 Ligases may act upon *REEP1*. These may, in turn, display different expression patterns in cortical and spinal motoneurons, leading to alternative turnover mechanisms. In summary, we propose that spinal motoneurons more easily accumulate variant *REEP1* protein compared to cortical motoneurons if its ubiquitination is compromised. The excess of the shaping‐competent variant then leads to the fragmentation of the ER structure, which compromises the long‐term survival of spinal motoneurons.

## Conflict of Interest

The authors declare no conflict of interest.

## Author Contributions

Initiation – C.B. and C.A.H. Conceptualization – I.D. and C.A.H. Writing – A.B., R.M.B., I.D., C.A.H. Methodology – S.N., O.H., J.W., M.M.K., R.M.B., B.Q., I.D., and C.A.H. Investigation – A.B., M.S., M.W., A.M., E.S., K.L., I.K., S.M., L.L., P.F., M.H.H., M.K., T.M., R.M.B. Analysis – A.B., M.S., M.W., A.M., E.S., K.L., I.K., S.M., L.L., P.F., M.H.H., M.K., T.M., R.M.B. Provision of patient data – T.L., M.d.V., and M.A.J.W. Supervision – M.K., O.H., J.W., M.M.K., R.M.B., B.Q., I.D., and C.A.H. Funding acquisition – I.D and C.A.H.

## Supporting information



Supporting Information

## Data Availability

The data that support the findings of this study are available from the corresponding author upon reasonable request.
