# Supplementary material for: REEP1 Accumulation Disrupts ER Integrity and Drives Spinal Motoneuron Degeneration in Distal Hereditary Motor Neuropathy
Source: Adv Sci (Weinh). 2025 Nov 21;13(2):e11483. doi: 10.1002/advs.202511483 (PMC12786278; doi:10.1002/advs.202511483)
Supplement: Supplementary file 1 — Supporting Information [file ADVS-13-e11483-s001.pdf]

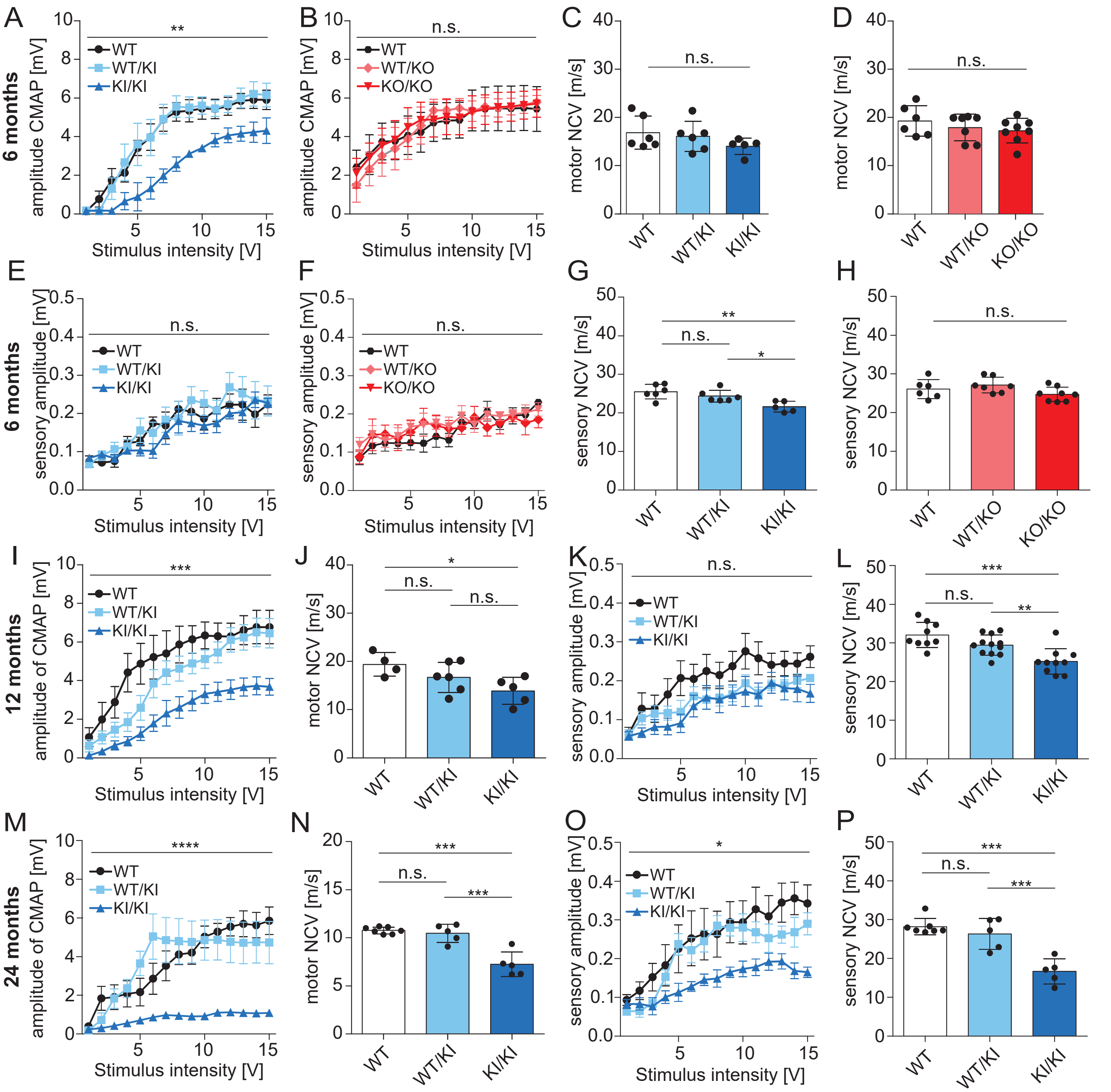

Figure S1. Electrophysiological analysis of WT, KO/KO, and KI/KI mice. A) Compound muscle action potential (CMAP) amplitudes of 6-month-old Reep1WT, Reep1WT/KI, and Reep1KI/KI (n = 6 WT, 6 WT/KI, and 5 KI/KI mice; two-way ANOVA; ** p<0.01). **B)** CMAP amplitudes of 6-month-old Reep1WT, Reep1WT/KO, and Reep1KO/KO mice (n = 6 WT, 7 WT/KO, and 8 KO/KO mice; two-way ANOVA; n.s.: not significant). **C)** Motor nerve conduction velocity (NCV) of 6-month-old Reep1WT, Reep1WT/KI, and Reep1KI/KI mice (n = 6 WT, 6 WT/KI, and 5 KI/KI mice; one-way ANOVA with Tukey's post-hoc test; n.s.: not significant). **D)** Motor NCVs of 6-month-old Reep1WT, Reep1WT/KO, and Reep1KO/KO mice (n = 6 WT, 7 WT/KO, and 8 KO/KO mice; one-way ANOVA with Tukey's post-hoc test; n.s.: not significant). **E)** Sensory amplitudes of 6-month-old Reep1WT, Reep1WT/KI, and Reep1KI/KI (n = 6 WT, 6 WT/KI, and 5 KI/KI mice; two-way ANOVA; n.s.: not significant). **F)** Sensory amplitudes of 6-month-old Reep1WT, Reep1WT/KO, and Reep1KO/KO mice (n = 6 WT, 7 WT/KO, and 8 KO/KO mice; one-way ANOVA with Tukey's post-hoc test; n.s.: not significant). **G)** Sensory NCV of 6-month-old Reep1WT, Reep1WT/KI, and Reep1KI/KI mice (n = 6 WT, 6 WT/KI, and 5 KI/KI mice; one-way ANOVA with Tukey's post-hoc test; * p<0.05; ** p<0.01). **H)** Sensory NCVs of 6-month-old Reep1WT, Reep1WT/KO, and Reep1KO/KO mice (n = 6 WT, 7 WT/KO, and 8 KO/KO mice; one-way ANOVA; n.s.: not significant). **I)** Compound muscle action potential (CMAP) amplitudes of 12-month-old Reep1WT, Reep1WT/KI, and Reep1KI/KI (n = 4 WT, 6 WT/KI, and 5 KI/KI mice; two-way ANOVA; *** p<0.001). **J)** Motor NCV of 12-month-old Reep1WT, Reep1WT/KI, and Reep1KI/KI mice (n = 7 WT, 5 WT/KI, and 5 KI/KI mice; one-way ANOVA with Tukey's post-hoc test; * p<0.05; n.s.: not significant). **K)** CMAP amplitudes of 24-month-old Reep1WT, Reep1WT/KI, and Reep1KI/KI (n = 7 WT, 5 WT/KI, and 5 KI/KI mice; two-way ANOVA; **** p<0.0001). **L)** Motor NCV of 24-month-old Reep1WT, Reep1WT/KI, and Reep1KI/KI mice (n = 7 WT, 5 WT/KI, and 5 KI/KI mice; one-way ANOVA with Tukey's post-hoc test; *** p<0.001; n.s.: not significant). **M)** Sensory amplitudes of 24-month-old Reep1WT, Reep1WT/KI, and Reep1KI/KI (n = 9 WT, 11 WT/KI, and 10 KI/KI mice; two-way ANOVA; n.s.: not significant). **N)** Sensory NCV of 24-month-old Reep1WT, Reep1WT/KI, and Reep1KI/KI mice (n = 9 WT, 11 WT/KI, and 10 KI/KI mice; one-way ANOVA with Tukey's post-hoc test; ** p<0.01; *** p<0.001; n.s.: not significant).**O)** Sensory amplitudes of 24-month-old Reep1WT, Reep1WT/KI, and Reep1KI/KI (n = 7 WT, 5 WT/KI, and 5 KI/KI mice; two-way ANOVA; * p<0.05). **P)** Sensory NCV of 24-month-old Reep1WT, Reep1WT/KI, and Reep1KI/KI mice (n = 7 WT, 5 WT/KI, and 5 KI/KI mice; one-way ANOVA with Tukey's post-hoc test; *** p<0.001; n.s.: not significant). Quantitative data are shown as mean ± SD.

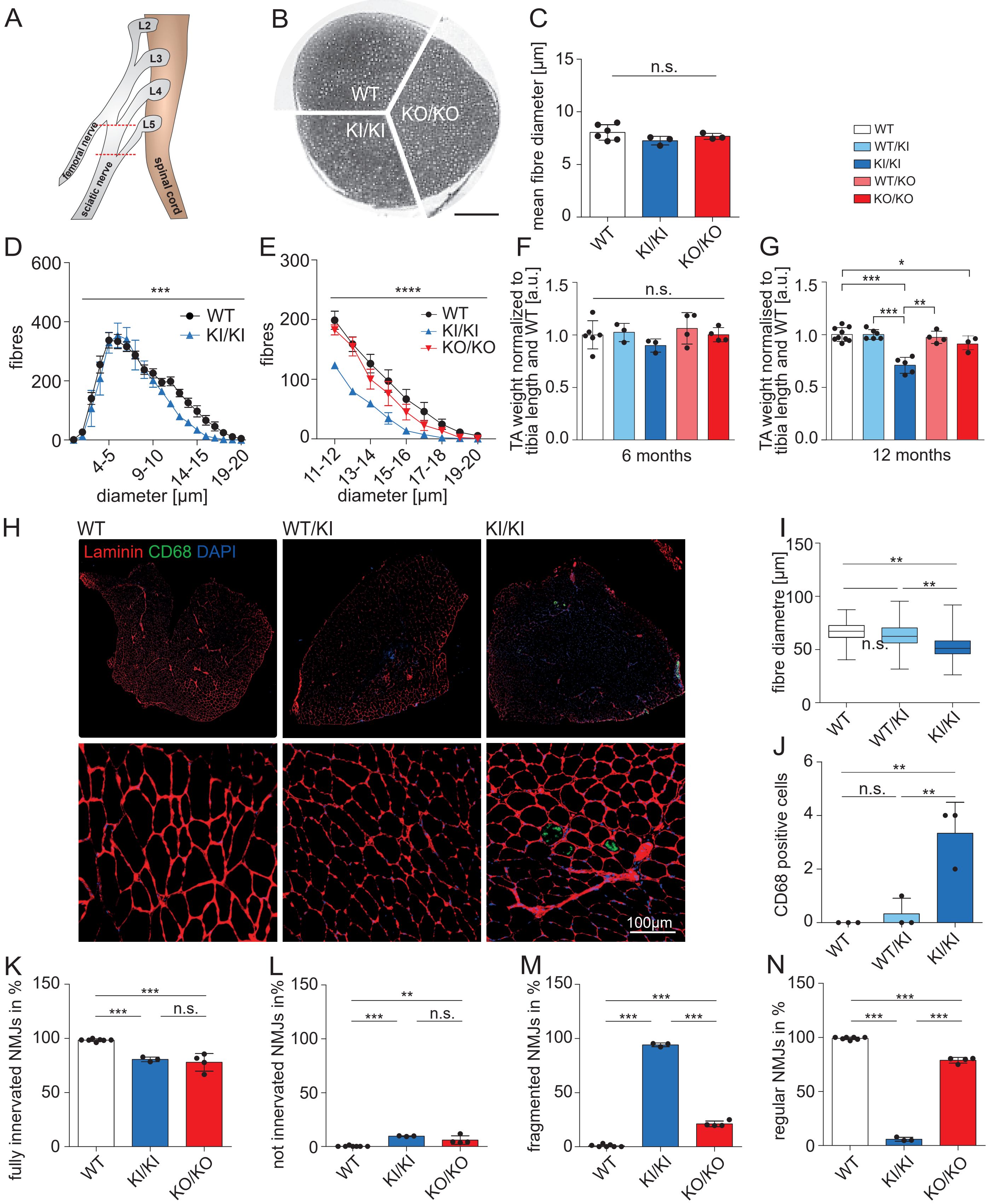

Figure S2. Decreased number of large diameter axons in KI/KI mice. A) Illustration of the lumbar spinal cord with femoral and sciatic nerve rootlets. The red dashed lines indicate the location of the sciatic nerve specimen assessed. **B)** Representative 0.5 µm semi-thin partial sciatic nerve cross sections stained with toluidine blue. Scale bar: 200 µm. **C)** Quantification of the mean fibre diameter (n=6 WT, 3 KO/KO, and 3 KI/KI mice; one-way ANOVA; n.s.: not significant). **D)** Distribution of axon diameters of Reep1 WT and Reep1 KI/KI mice at 12 months of age (n = 6 WT and 3 KI/KI mice; one-way ANOVA; ** p<0.01). **E)** Distribution of larger diameter axons of Reep1 WT, Reep1 KI/KI, and Reep1 KO/KO mice (n = 6 WT, 3 KO/KO, and 3 KI/KI mice; one-way ANOVA; **** p<0.0001). **F)** Weight of the Musculus tibialis anterior normalized to tibia length at 6 months of age (n = 6 WT, 3 WT/KI, 3 KI/KI, 4 WT/KO, and 4 KO/KO mice; one-way ANOVA with Tukey's post-hoc test). **G)** Weight of the Musculus tibialis anterior normalized to tibia length at 12 months of age (n = 9 WT, 6 WT/KI, 5 KI/KI, 4 WT/KO, and 3 KO/KO mice; one-way ANOVA with Tukey's post-hoc test; * p<0.05 ** p<0.01; *** p<0.001). **H)** Staining of Musculus tibialis anterior cross sections for Laminin (sarcolemma, red) and CD68 (skeletal muscle macrophages, green). **I)** The homozygous deletion of exon 5 decreases mean skeletal muscle fibre diameters (n = 3; one-way ANOVA with Tukey's post-hoc test; *** p<0.001). **J)** The number of skeletal muscle macrophages is increased in 12-month-old Reep1 KI/KI mice (n = 3; one-way ANOVA; *** p<0.001). **K-N)** Quantification of fully innervated (K), not innervated (L) fragmented (M), and regular neuromuscular junctions (NMJ) (N) (n = 7 WT, 4 KO/KO, and 3 KI/KI mice; one-way ANOVA with Tukey's post-hoc test). Quantitative data are shown as mean ± SD.

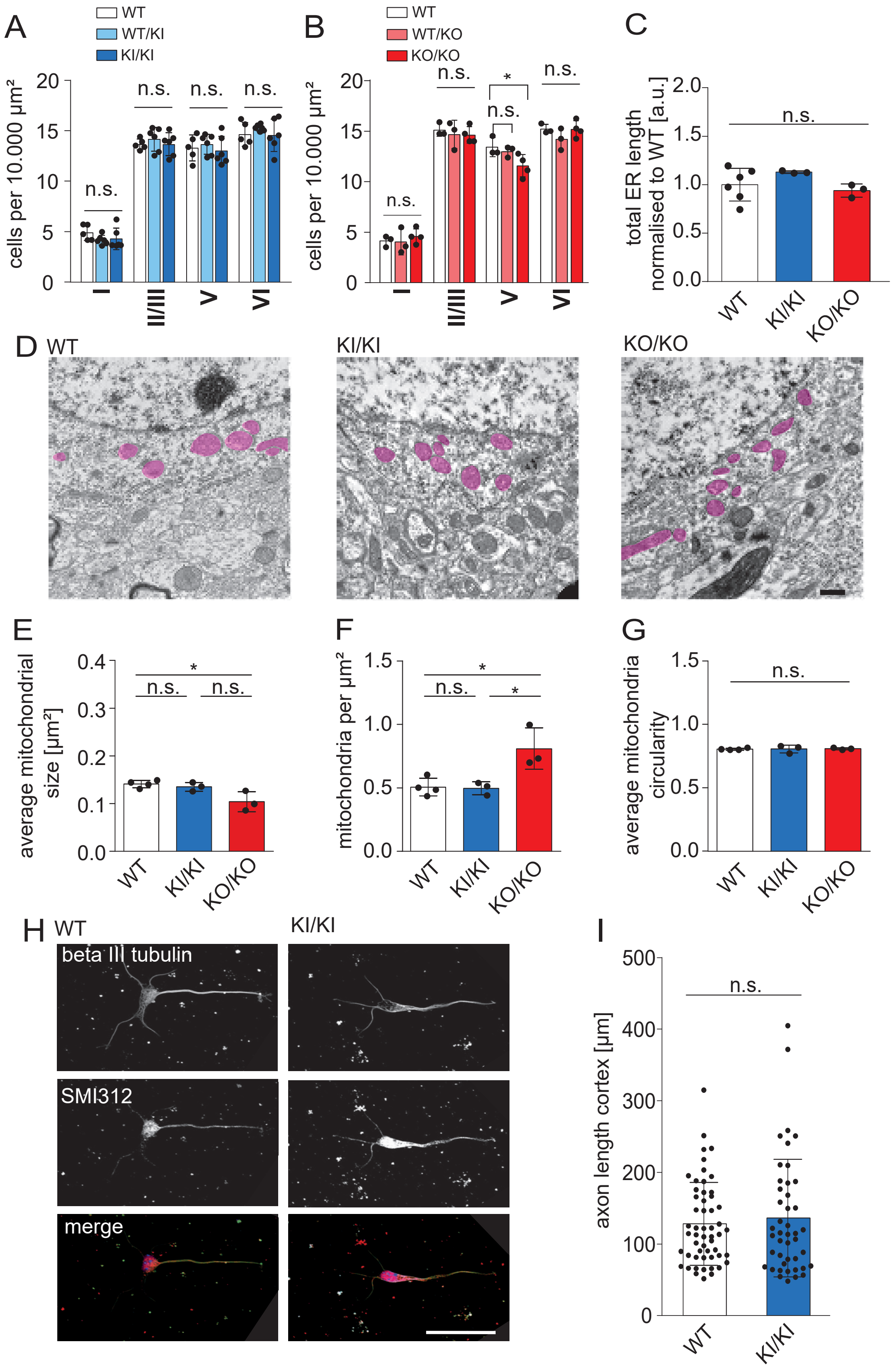

Figure S3. Analysis of cortical motoneurons in WT, KO/KO, and KI/KI mice. A) Quantification per layer for WT, WT/KI, and KI/KI mice (n = 5 WT, 6 WT/KI, and 6 KI/KI mice; two-way ANOVA with Bonferroni post-hoc test). **B)** Quantification per layer WT, WT/KO, and KO/KO mice (n = 3 WT, 3 WT/KO, and 4 KO/KO mice; two-way ANOVA with Bonferroni post-hoc test. * $p < 0.05$). **C-G)** Ultrastructural analysis of cortical motoneurons. C) Total ER length normalized to the cell area and WT. Data points represent individual animals (n = 6 WT, 3 KI/KI and 3 KO/KO mice; one-way ANOVA with Tukey's post-hoc test: * $p<0.05$, ** $p<0.01$, *** $p<0.001$). D) Representative TEM images with mitochondria labeled in magenta (magnification: 3,000x) of 60 nm ultra-thin sections of M1 layer V of 12-month-old WT, KOI/KI, and KO/KO mice. Scale bar: 500 nm. Quantification of mitochondrial size (E), the number of mitochondria per cell area (F) and their circularity (G) (one-way ANOVA with Tukey's post-hoc test; * $p<0.05$). **H)** Representative images of cultured cortical primary neurons isolated from P1 WT and KI/KI pups. After 3 days in vitro, neurons were stained for beta III Tubulin and the axon marker SMI312, and the length of the longest SMI312-positive protrusion (axon) was assessed. Scale bar: 50 µm. **I)** Quantification of axon outgrowth from n = 54 WT and 44 KI/KI neurons (unpaired Student's t-test). Quantitative data are shown as mean ± SD.

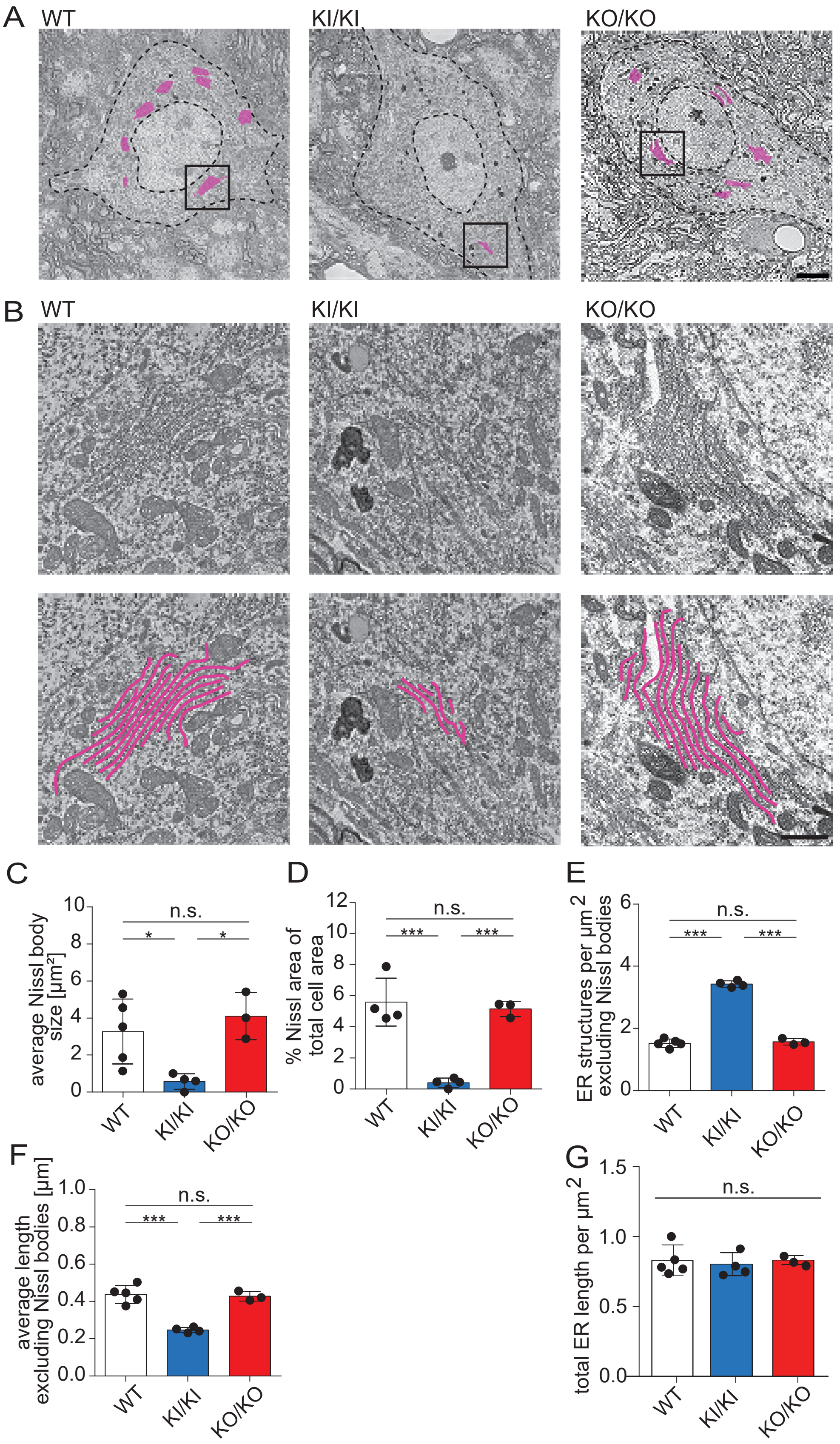

Figure S4. Ultrastructural analysis of spinal motoneurons in WT, KI/KI, and KO/KO mice.
A) Representative TEM images (magnification: 4,000x) of 60 nm ultra-thin sections of spinal motor neurons from a 12-month-old WT, KO/KO, and KI/KI mouse. Nissl bodies are marked in magenta. Scale bar: 5 µm. **B)** Magnifications of the regions indicated in A (magnification: 12,000x). In the lower panel, Nissl bodies are marked in magenta. Scale bar: 1 µm. **C, D)** Quantification of the average size of Nissl bodies per cell (C) and the area covered by Nissl bodies per cell (D). **E, F)** Number (E) and average length (F) of individual ER structures excluding Nissl bodies. **G)** Cumulative length of ER structures excluding Nissl bodies. Each data point represents the mean of ≥ 3 cells of one individual animal. ≥ 3 cells per animal from n = 5 WT, 3 KO/KO, and 4 KI/KI mice; one-way ANOVA with Tukey's post-hoc test: *** p<0.001. Quantitative data are shown as mean ± SD.

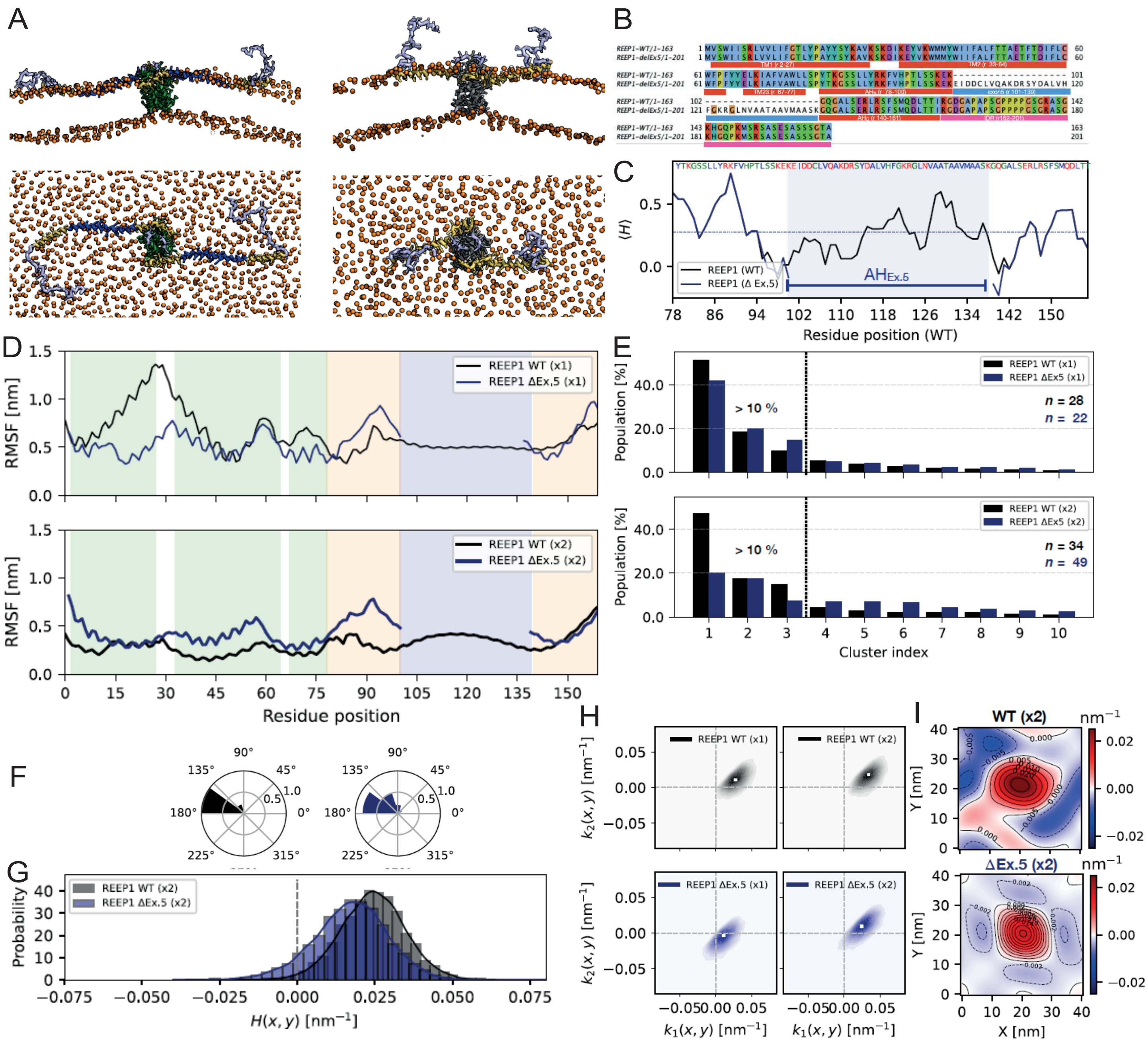

Figure S5. Modeling and simulations of REEP1 dimers. A) Representative snapshots (top and side views) of REEP1 dimers, WT (left) and the exon5-deletion (right) from 10 µs long MD simulations. The TM segments (green) and the AH segments (yellow), along with the region encoded by exon 5 (blue), anchor the protein in the POPC bilayer (PO4 groups in orange). Note the antiparallel arrangement of the AH segments in both proteins. **B)** Jalview rendering of the sequence alignment (colored according to the Clustal scheme) and annotation of the TM and AH segments of REEP1 WT and exon5-deletion. **C)** Average hydrophobicity ⟨H⟩ along the length for the WT AH segment (⟨H⟩ = 0.27 ± 0.17) and the exon5-deletion AH segment (⟨H⟩ = 0.27 ± 0.22). D) Root mean square fluctuations (RMSF) plots relative to the average structure of WT and Δexon5 for monomeric REEP1 (top) and dimeric REEP1 variants (bottom). **E)** Cluster analysis showing the top 10 clusters of monomeric (top) and dimeric (bottom) REEP1 variants, showing the distribution of distinct conformational states (RMSD > 0.5 nm). Only the top 3 clusters have a population > 10% population, and n denotes the number of clusters obtained. **F)** Circular histograms of angles between AH segments of the two protomers of the dimeric WT and exon5-deletion variant, sampled from MD simulations, show a relative asymmetry of induced curvature fields. **G)** Probability densities (and histograms) of intrinsic mean curvature sampled by the dimeric WT protein (H(x,y) = 0.024 nm-1) and exon5-deletion variant (H(x,y) = 0.017 nm-1). **H)** 2D histograms of the principal membrane curvatures (k_1 and k_2) sampled by monomeric (left) and dimeric (right) REEP1, WT (top), and Δexon5 (bottom). White circles mark the most probable values. **I)** Protein-induced mean curvature fields illustrating local membrane perturbation due to dimeric WT (top) and Δexon5 (bottom).

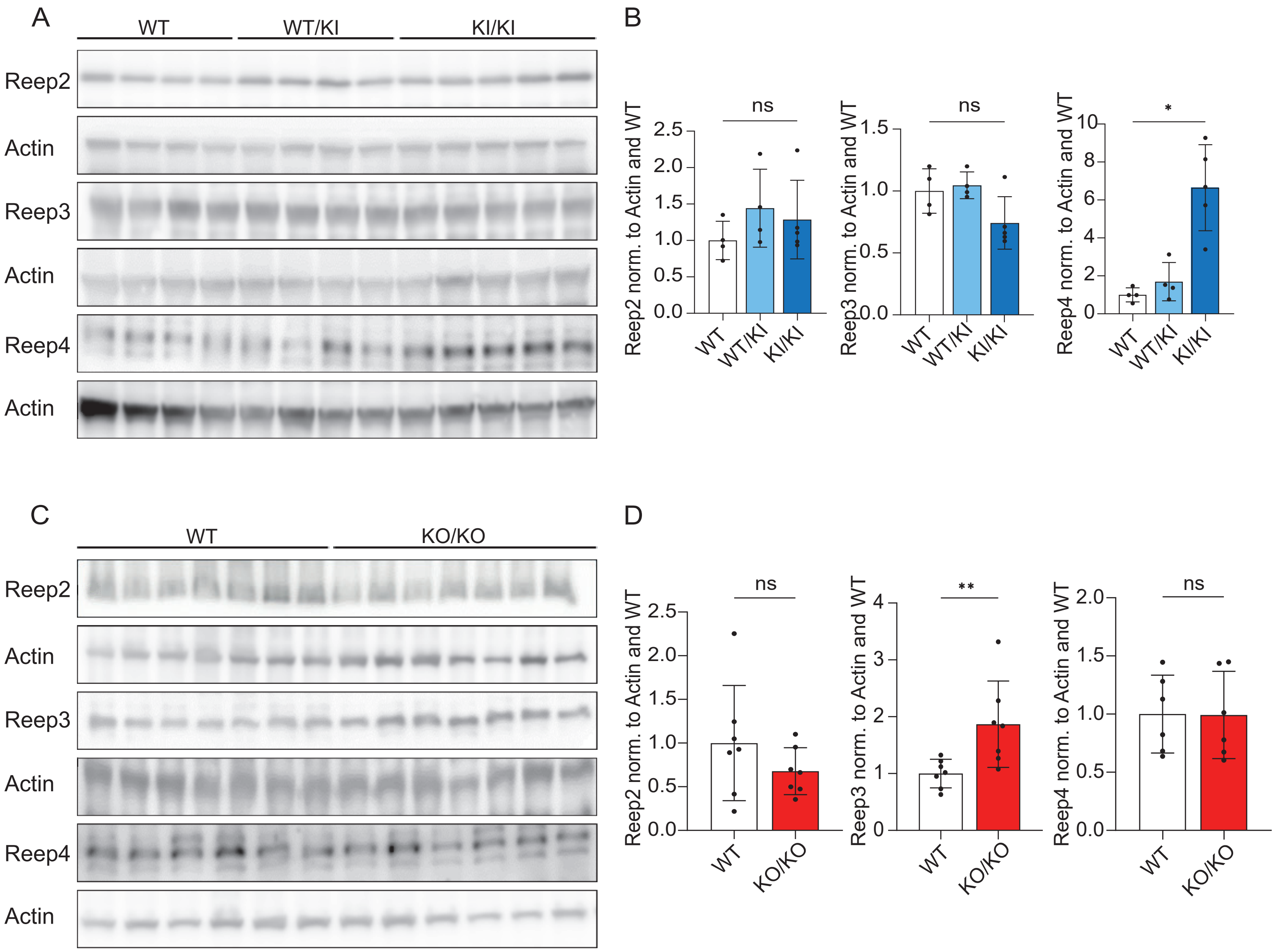

Figure S6. Abundances of Reep2, Reep3, and Reep4 in brain and spinal cord protein lysates. **A)** Immunoblot of Reep2, Reep3, and Reep4 in spinal cord lysates from 2-month-old WT, WT/KI, and KI/KI mice. **B)** Quantification normalised to Actin and WT (n = 4 WT, 4 WT/KI, and 5 KI/KI mice; Kruskal-Wallis test with Dunn`s post-hoc test; * $p<0.05$; n.s.: not significant). **C)** Immuno-blot of Reep2, Reep3, and Reep4 in brain lysates from 12-month-old WT and KO/KO mice. **D)** Quantification normalised to actin and WT (Reep2 and Reep3 n = 7 WT and 7 KO/KO mice; Reep4 n = 6 WT and 6 KO/KO mice; Mann-Whitney U test; ** $p<0.01$; n.s.: not significant). Quantitative data are shown as mean ± SD.

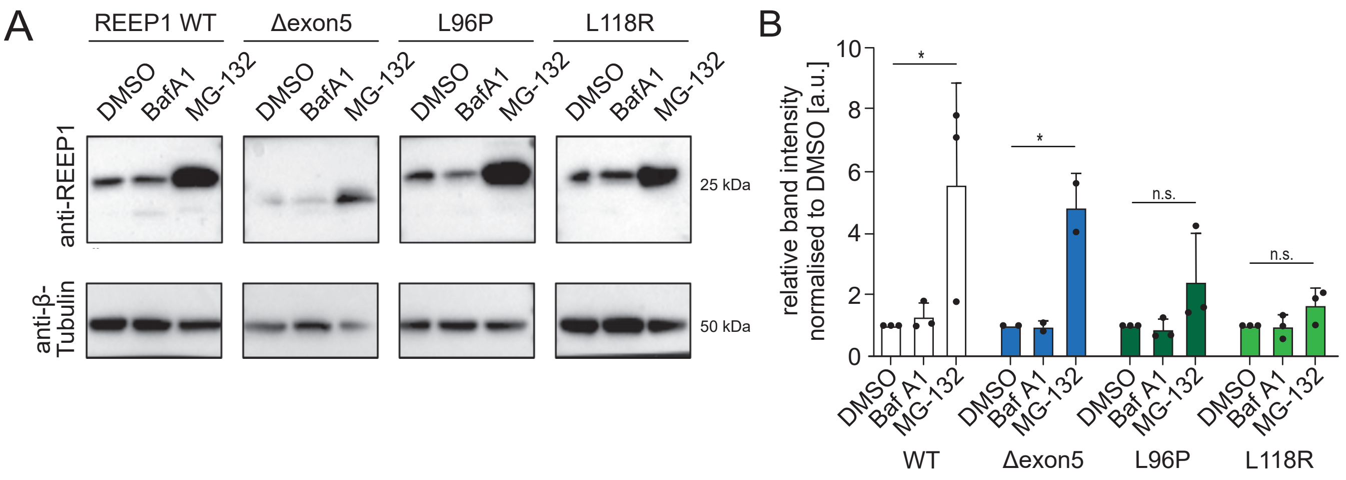

C

REEP1 WT

aa	log2 fold change	-log p-value	T-test significant?
K90	2.98	2.97	+
K99	2.54	4.81	+
K123	2.66	2.18	+
K139	2.63	4.91	+

P19L

aa	log2 fold change	-log p-value	T-test significant?
K90	4.75	1.44	+
K99	2.27	0.90	-
K123	3.30	1.53	+
K139	1.76	1.44	+

T55K

aa	log2 fold change	-log p-value	T-test significant?
K90	4.58	4.34	+
K99	2.30	1.88	+
K123	3.66	4.66	+
K139	0.61	0.63	-

Δexon5

aa	log2 fold change	-log p-value	T-test significant?
K90	2.73	2.68	+
K99	2.62	2.31	-
K123	-	-	-
K139	-	-	-

L96P

aa	log2 fold change	-log p-value	T-test significant?
K90	-	-	-
K99	-	-	-
K123	2.34	1.91	-
K139	2.18	3.12	-

L118R

aa	log2 fold change	-log p-value	T-test significant?
K90	2.74	2.63	+
K99	2.34	3.59	+
K123	-	-	-
K139	1.88	0	-

(K123 and K139: Exon 5)

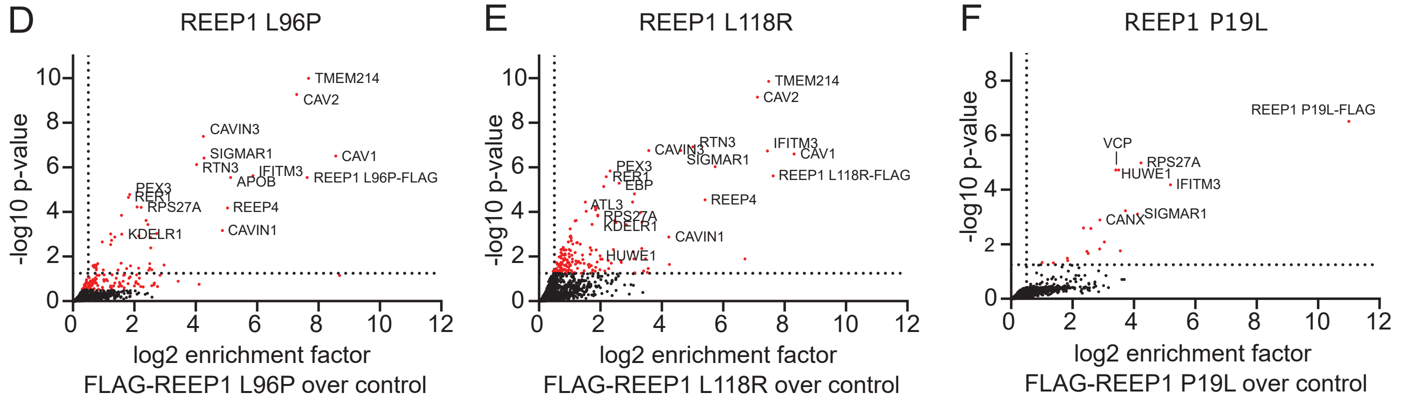

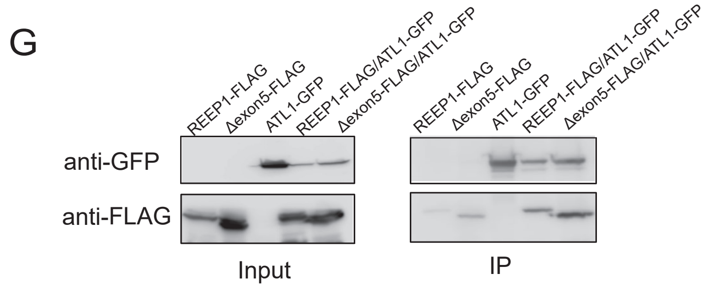

Figure S7. Ubiquitination profile of REEP1 variants and additional interactomes. A, B) The REEP1 WT and the dHMN-associated variants Δexon5, L96P and L118R are degraded via the proteasomal system and not by autophagy. Quantification of REEP1 WT in stably transfected HeLa-TRex cells upon either inhibition of the proteasome with MG-132 or inhibition of autophagy with Bafilomycin A1 (one-way ANOVA with Tukey's post-hoc test; * $p<0.05$). The data for REEP1 WT are also displayed in Figure 7A and B. **C)** Ubiquitination sites of REEP1 and its variants were determined through mass spectrometry analysis of the interactomes, which quantified GlyGly-modified peptides from tryptic digestion and assigned them to REEP1. The significance of the ubiquitination site was determined using Student's t-test; significant sites are highlighted in red. n=3 biological replicates per variant. **D-F)** Mass spectrometry analysis of HeLa Trex cells induced with doxycycline expressing REEP1 L96P-FLAG (D), REEP1 L118R-FLAG (E), or REEP1 T55K-FLAG (F) compared to HeLa-TRex parental cells. N = 3 biological replicates per variant, significance cut-off placed at -log10 p-value of 1.25, log2 Fold Change of 0.5. **G)** Both REEP1 WT and the Δexon5 interact with ATL1. REEP1-FLAG or Δexon5-FLAG and ATL1-GFP were heterologously expressed in HEK-293T cells (representative of n = 2 independent experiments). Quantitative data are shown as means ± SD.

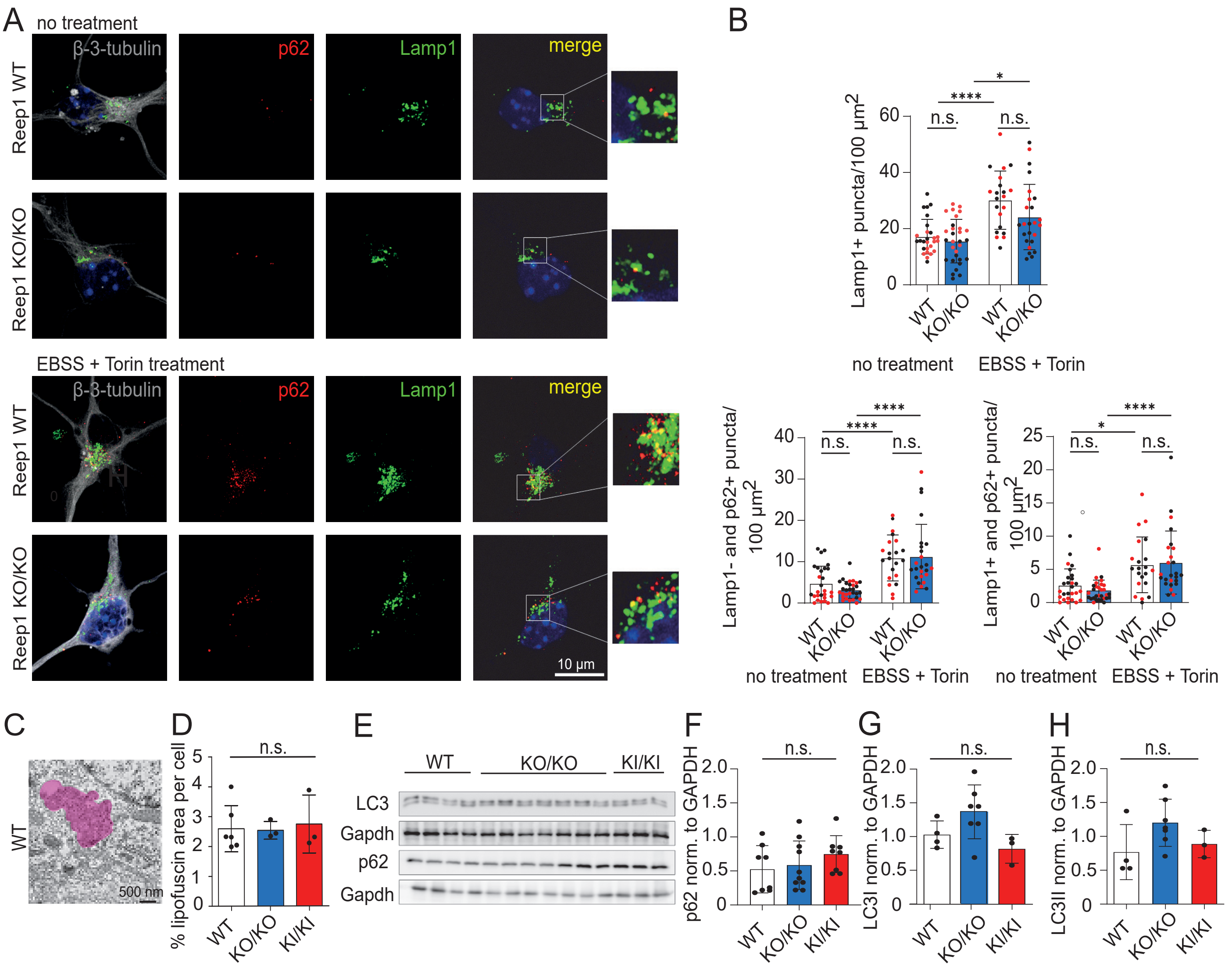

Figure S8. The KO of Reep1 does not impair autophagy. A, B) Autophagy was induced by EBSS starvation in addition to Torin1 in cultured primary cortical neurons from WT and KO/KO pups. After 8 h, the cells were fixed and stained for p62/Sqstm1, Lamp1, and β3-Tubulin to identify neurons. Autophagosomes, defined as p62-positive and Lamp1-negative puncta, and autolysosomes, defined as puncta positive for both p62 and Lamp1, did not differ at steady state and upon induction of autophagy between genotypes (one-way ANOVA, n = 2 experiments with 15 neurons each). **C, D)** The area of lipofuscin particles in cortical motoneurons did not differ between genotypes in TEM of cortical motoneurons (one-way ANOVA with 7 cells each from 5 WT, 3 KO/KO, and 3 KI/KI mice). **E-H)** The abundances of p62 and LC3B-II normalized to GAPDH did not differ in WT, KO/KO, and KI/KI brain lysates (one-way ANOVA with samples from 8 WT, 10 KO/KO, and 8 KI/KI mice). Quantitative data are shown as mean ± SD.
